# Structural Elucidation of Ivermectin Binding to α7nAChR and the Induced Channel Desensitization

**DOI:** 10.1021/acschemneuro.2c00783

**Published:** 2023-02-23

**Authors:** Vasyl Bondarenko, Qiang Chen, Kevin Singewald, Nandan Haloi, Tommy S. Tillman, Rebecca J. Howard, Erik Lindahl, Yan Xu, Pei Tang

**Affiliations:** † Department of Anesthesiology and Perioperative Medicine, University of Pittsburgh, Pittsburgh, Pennsylvania 15260, United States; ‡ Department of Chemistry, University of Pittsburgh, Pittsburgh, Pennsylvania 15260, United States; § Department of Biochemistry and Biophysics, Science for Life Laboratory, Stockholm University, PO Box 1031, SE-17121 Solna, Sweden; ∥ Department of Applied Physics, Swedish e-Science Research Center, KTH Royal Institute of Technology, PO Box 1031, SE-17121 Solna, Sweden; ⊥ Department of Pharmacology and Chemical Biology, University of Pittsburgh, Pittsburgh, Pennsylvania 15260, United States; # Department of Structural Biology, University of Pittsburgh, Pittsburgh, Pennsylvania 15260, United States; ∇ Department of Physics and Astronomy, University of Pittsburgh, Pittsburgh, Pennsylvania 15260, United States; ○ Department of Computational and Systems Biology, University of Pittsburgh, Pittsburgh, Pennsylvania 15260, United States

**Keywords:** ivermectin binding, α7 nicotinic acetylcholine receptor, α7nAChR, channel desensitization, NMR, modeling

## Abstract

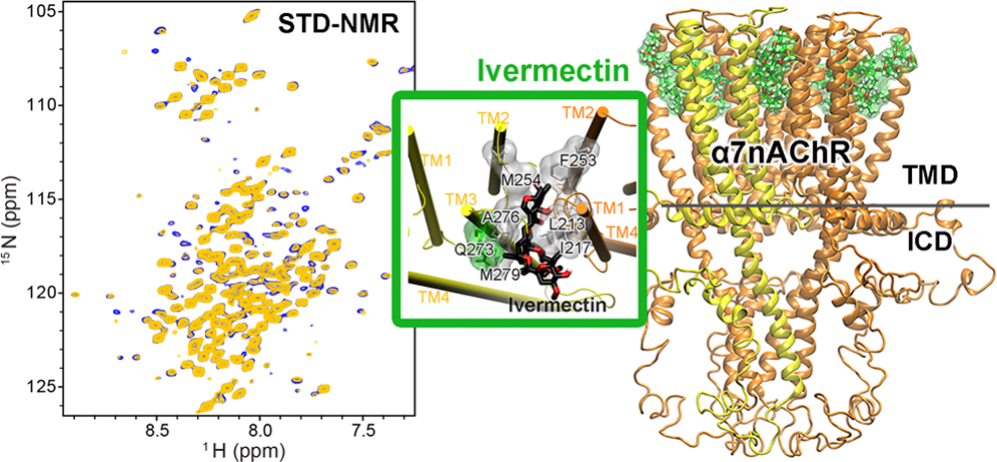

The α7 nicotinic acetylcholine receptor (α7nAChR) mediates signaling in the central nervous system and cholinergic anti-inflammatory pathways. Ivermectin is a positive allosteric modulator of a full-length α7nAChR and an agonist of the α7nAChR construct containing transmembrane (TMD) and intracellular (ICD) domains, but structural insights of the binding have not previously been determined. Here, combining nuclear magnetic resonance as a primary experimental tool with Rosetta comparative modeling and molecular dynamics simulations, we have revealed details of ivermectin binding to the α7nAChR TMD + ICD and corresponding structural changes in an ivermectin-induced desensitized state. Ivermectin binding was stabilized predominantly by hydrophobic interactions from interfacial residues between adjacent subunits near the extracellular end of the TMD, where the inter-subunit gap was substantially expanded in comparison to the apo structure. The ion-permeation pathway showed a profile distinctly different from the resting-state profile but similar to profiles of desensitized α7nAChR. The ICD also exhibited structural changes, including reorientation of the MX and h3 helices relative to the channel axis. The resulting structures of the α7nAChR TMD + ICD in complex with ivermectin provide opportunities for discovering new modulators of therapeutic potential and exploring the structural basis of cytoplasmic signaling under different α7nAChR functional states.

## Introduction

The α7 nicotinic acetylcholine receptor (α7nAChR) is a major subtype of neuronal nAChRs in the brain that forms pentameric channels with high Ca^2+^ permeability^1^ and mediates critical functions in the central nervous system.^2^ α7nAChR is also a major player in the cholinergic anti-inflammatory pathway,^3,4^ for which anti-inflammatory activities of α7nAChR in non-neuronal cells are mainly mediated through interactions of the intracellular domain (ICD) with cytoplasmic proteins.^5,6^ The involvement of α7nAChR in a broad spectrum of physiological and pathological processes has made this receptor an attractive target for the treatment of various disorders and diseases, such as inflammation and pain,^5,7^ Alzheimer’s disease,^8^ schizophrenia,^9^ and addiction.^10^

Like other members in the Cys-loop receptor family, α7nAChR exhibits distinct conformational changes as it cycles among three major functional states: resting, activation, and desensitization. Functional-dependent conformational changes in the extracellular domain (ECD) and transmembrane domain (TMD) of α7nAChR have been well captured in cryo-EM structures.^11,12^ These structures demonstrate a gating cycle from a closed-pore conformation in a resting state (PDBIDs: 7EKI, 7KOO) to an open-pore conformation of α7nAChR (PDBIDs: 7EKT, 7KOX) by co-binding of agonist and the positive allosteric modulator PNU-120596, and further to a closed-pore conformation in a desensitized state (PDBIDs: 7EKP, 7KOQ) when an agonist alone was bound. It is noteworthy that similar state-dependent conformational changes were observed, including the profiles of the ion-permeation pathway, when channel desensitization was induced by different α7nAChR agonists.^11,12^ In contrast to the comprehensive information about the ECD and TMD of α7nAChR, the ICD structure and its changes over the transition from the resting to a desensitized state are only partially resolved in the cryo-EM structures,^11,12^ largely due to the intrinsic flexibility of the ICD. Recently, we determined the full-length ICD structures of the human α7nAChR in the resting state through combining nuclear magnetic resonance (NMR) and electron spin resonance (ESR) with Rosetta comparative modeling that uses the experiments as restraints.^13^

Ivermectin (IVM) is a well-known antiparasitic drug that kills parasites by activating glutamate-gated Cl^–^ channel receptors (GluClRs). It also targets P2X_4_ receptors, farnesoid X receptors, the G-protein-gated inwardly rectifying K^+^ channel, and some Cys-loop receptors.^14^ IVM potentiates the Cl^–^ -conducting function of GluClRs and glycine receptors (GlyRs).^15−18^ At high concentrations, it activates certain GABA_A_Rs and GlyRs.^19−21^ Structures of Cys-loop receptors in complex with IVM, including *Caenorhabditis elegans* GluCl,^15^ human α3GlyR,^16^ and zebrafish α1GlyRs,^17,18^ have been solved. These structures show IVM binding to an inter-subunit site in the TMD. IVM is also a positive allosteric modulator (PAM) of α7nAChR, and its preapplication strongly enhances agonist-evoked currents.^22−24^ In the absence of the ECD, IVM can even act as an agonist to elicit channel currents of *Xenopus* oocytes injected with the purified α7nAChR TMD^25^ or TMD + ICD^13^ that forms ion channels. These results also suggest the likelihood of IVM binding to the TMD of α7nAChR, but the exact action site of IVM in α7nAChR has previously not been determined.

Here, we reveal molecular details of IVM binding to the human α7nAChR TMD + ICD, an α7nAChR construct whose functions and structures in the resting state have previously been determined by combining NMR, ESR experiments, and Rosetta modeling.^13^ Using a similar approach with additional molecular dynamics (MD) simulations, we have determined not only structural insights of IVM binding but also structural changes of the α7nAChR TMD + ICD in a desensitized state induced by IVM. The resulting structures of the α7nAChR TMD + ICD in complex with IVM provide opportunities for discovering new α7nAChR modulators of therapeutic potential and exploring the structural basis of cytoplasmic signaling under different α7nAChR functional states.

## Results and Discussion

### IVM-Induced Desensitization of the α7nAChR TMD+ICD

**Figure 1:**
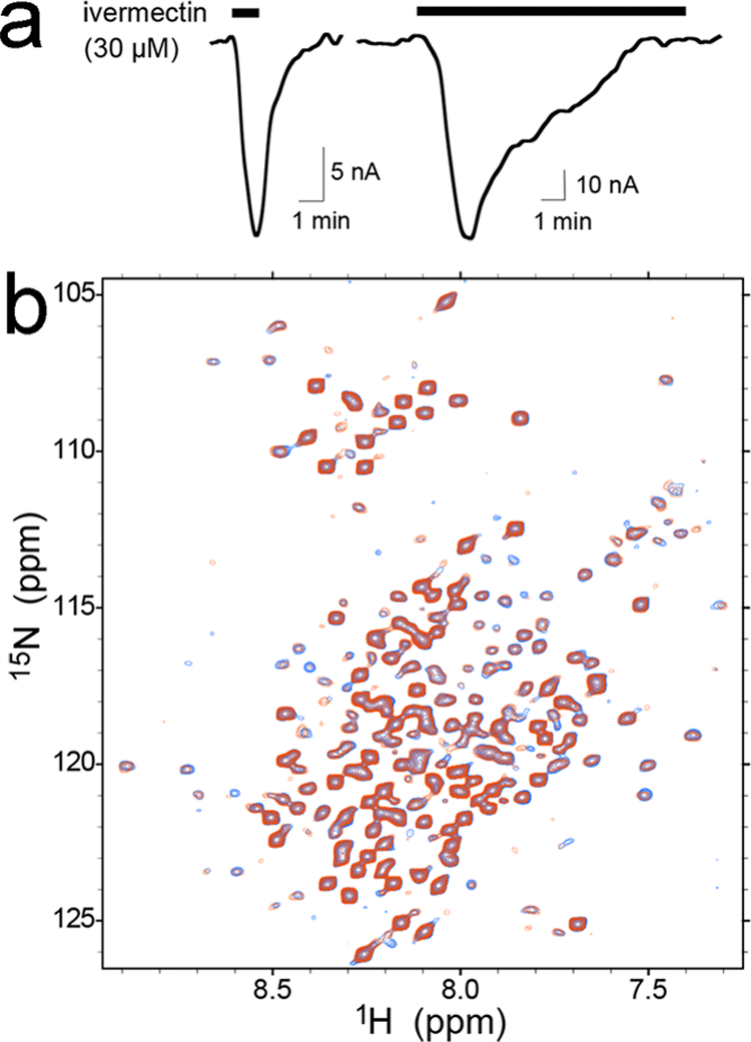
Desensitization of the α7nAChR TMD+ICD. (a) Current traces of *Xenopus* oocytes injected with purified TMD + ICD reconstituted into asolectin vesicles, showing channel activation by ivermectin (left) and desensitization by prolonged application of ivermectin (right). (b) Overlay of representative ^1^H-^15^N TROSY-HSQC NMR spectra of α7nAChR TMD + ICD in LDAO micelles in the absence (blue) and presence (red) of 30 μM ivermectin. The spectra were collected on an 18.8-tesla NMR spectrometer at 45°C. Additional chemical shift changes of the TMD + ICD from the resting to a desensitized state induced by different concentrations of ivermectin can be found in Supporting Information Figure S1.

We have previously reported a human α7nAChR construct containing the TMD and ICD (TMD + ICD) that is suitable for solution NMR structural studies.^13^ The TMD + ICD forms pentameric channels that can be activated by IVM and potentiated by the α7nAChR-specific PAM PNU-120596 in a concentration-dependent manner.^13^ It is known that IVM enhances agonist-evoked currents of the full-length α7nAChR but cannot activate the channel.^22,23^ Interestingly, in the absence of the ECD, IVM acts as an agonist, eliciting currents in *Xenopus* oocytes injected with purified α7nAChR TMD^25^ or TMD + ICD^13^ channels. Here, we find that the IVM-induced channel current of *Xenopus* oocytes injected with purified α7nAChR TMD + ICD can disappear in <10 min during prolonged IVM application (Figure 1a), an indication of channel desensitization. Correspondingly, IVM-induced desensitization of the TMD + ICD can be detected by NMR experiments, as demonstrated by chemical shift perturbations upon IVM binding (Figure 1b). Most residues experiencing chemical shift changes (>10 ppb) are in the TMD, particularly in the TM1, TM2, and TM3 helices, but changes in the ICD residues are also notable (Supporting Information Figures S1 and S2). To differentiate whether the observed chemical shift changes result from direct perturbation of IVM binding to its contacting residues or conformation changes distal from the IVM binding site, we have performed different NMR experiments as described below. It is noteworthy that ivermectin can act not only as a PAM of α7nAChR but also as an agonist of the α7nAChR TMD + ICD. The finding suggests that the ECD is not merely for inducing gating upon agonist binding, it also has a role in stabilizing the resting state of α7nAChR. This notion is also supported by a previous study, which shows spontaneous opening of α1GlyR in the absence of the ECD.^26^

### IVM Binding Site in α7nAChR

**Figure 2:**
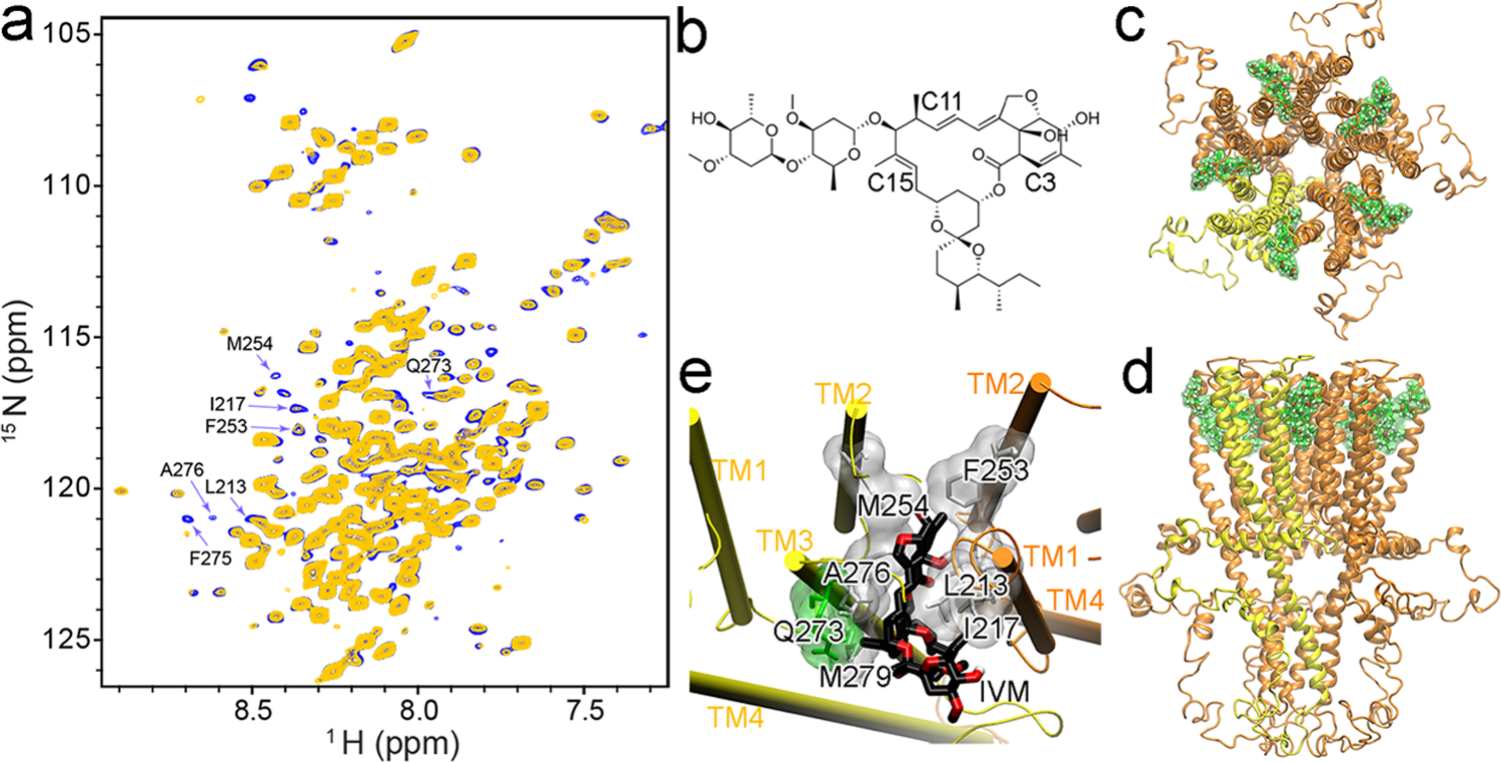
Ivermectin binding site in the α7nAChR TMD+ICD. (a) 2D saturation transfer (STD) NMR spectra of the α7nAChR TMD + ICD with (yellow) and without (blue) saturation of IVM C11 proton. Residues displaying profound peak intensity changes are marked and used in HADDOCK. Spectra with ^1^H saturation at other IVM positions are presented in Supporting Information Figures S3, S4. (b) IVM chemical structure marked with carbons saturated in STD NMR experiments. (c) Top view and (d) side view of a representative desensitized α7nAChR TMD + ICD structure showing IVM (green surface) bound to an inter-subunit cleft in the TMD. (e) A zoom-in view of IVM binding to a cleft between two adjacent subunits colored in yellow and orange. The IVM-contacting residues are colored according to the residue type: nonpolar—white and polar—green.

It has been suggested previously that IVM would act in the TMD of α7nAChR,^22,23^ but structural details of the IVM binding have not previously been elucidated. To determine where IVM binds in α7nAChR, we performed heteronuclear two-dimensional (2D) saturation transfer difference (STD) NMR,^25,27^ which provides information on the intermolecular interface of the α7nAChR-IVM complex by cross-saturation from IVM to α7nAChR. ^1^H signals of IVM were selectively saturated (Supporting Information Figure S3) and then cross-relaxed to the α7nAChR residues in close contact to IVM, resulting in a reduction of peak intensities in the measured ^1^H-^15^N STD NMR spectra (Figure 2a, Supporting Information Figure S4). ^1^H saturation at C11 or C3 near or on the benzofuran ring affected more residues than saturating ^1^H-C15 in the STD spectra (Figure 2a,b, Supporting Information Figure S4). These STD experiments with different ^1^H saturation sites identified IVM-contacting residues and provided experimental restraints to guide HADDOCK,^28,29^ which presents IVM binding at a cleft between two adjacent subunits of α7nAChR proximal to the extracellular side of the membrane surface (Figure 2c–e).

In the α7-IVM complex structures, IVM is in close contact (≤ 3.0 Å) with residues L213, I217, and P218 in TM1 and F253 in TM2 from the complementary (−) subunit, and the TM3 residues Q273, A276, M279 and the TM2 residue M254 from the principal (+) subunit (Figure 2e). Most of these residues are hydrophobic, highlighting the importance of hydrophobic interactions in IVM binding at this site. The insertion of IVM deep into the binding pocket is supported by strong STD effects observed on M254 (+) and F253 (−) when ^1^H on or near the benzofuran ring of IVM was selectively saturated. This IVM orientation resembles that observed in structures of GlyR-IVM and GluClR-IVM complexes,^15−18^ in which IVM has also placed its benzofuran ring deep into the inter-subunit binding site, the disaccharide outside the binding cleft, and the spiroketal moiety toward the cytoplasm. Comparing the IVM-contacting residues in α7nAChR to corresponding aligned regions in GlyRs and GluClR, there is 30–40% sequence identity and more than 60% sequence similarity (Supporting Information Table S1). The IVM C5-hydroxyl was reported previously as an essential molecular element for activation of α1GlyR^30^ and formed a hydrogen bond with α3GlyR S267.^16^ In α7nAChR, the equivalent residue to α3GlyR S267 is M254 (Supporting Information Table S1), to which the IVM C5-hydroxyl cannot form a hydrogen bond, though it has close contact (<3 Å) with the sulfur atom of M254. Interactions of IVM with M254 could influence IVM actions, as suggested in a previous mutagenesis study that showed M254L converted IVM from a PAM into an antagonist.^23^

The IVM binding site almost overlaps with the site for the PAM PNU-120596 shown in the cryo-EM structure of an α7nAChR complex (PDBID: 7EKT).^12^ However, neither of the two previously published desensitized α7nAChR structures (PDBIDs: 7KOQ, 7EKP)^11,12^ presents an inter-subunit space sufficiently large for IVM binding. Our α7nAChR-IVM complex structures show a wider inter-subunit interfacial space near the extracellular end of the TMD as described below.

### Structures of the α7-IVM Complex

Pentameric structures of the α7nAChR TMD + ICD in complex with IVM (in short, α7-IVM) were obtained using an iterative protocol^13^ with structure restraints derived from NMR and ESR experiments (Supporting Information Figures S1–S7, Supporting Information Table S2). The final α7-IVM structures were validated by independent restraints that had not been used in structural calculations (see the Methods section). The structure quality and statistics are summarized in Supporting Information Table S3. A bundle of the 15 lowest-energy α7-IVM structures deposited to PDB (PDBID: 8F4V) is presented in Supporting Information Figure S8.

**Figure 3:**
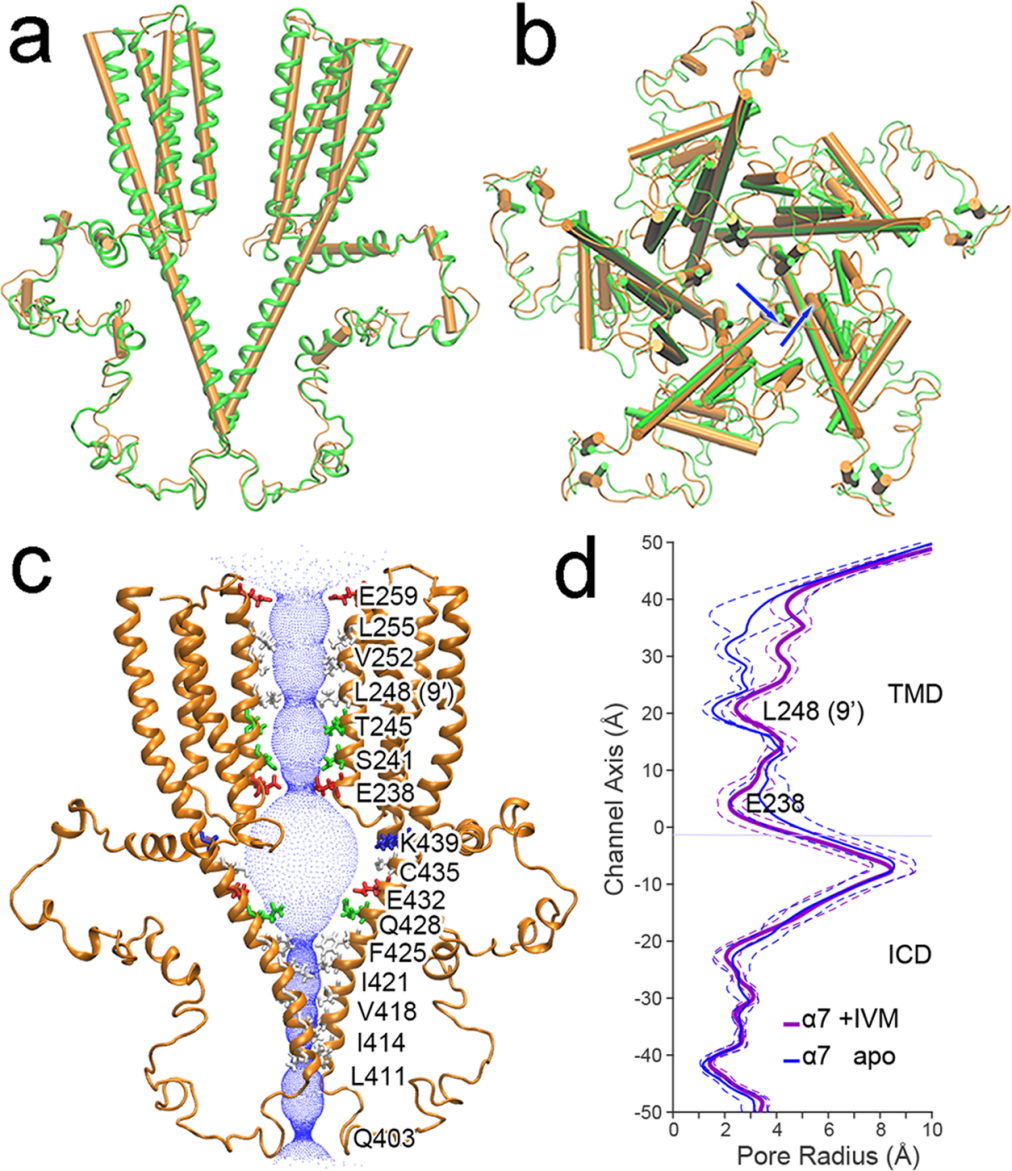
Structures of the α7nAChR TMD+ICD in the IVM-induced desensitization state. (a) Side view and (b) top view of aligned α7 TMD + ICD structures in the IVM-induced desensitized state (orange, PDBID: 8F4V) and resting state (green, PDBID: 7RPM). For clarity, only two subunits are shown in panel (a). The blue arrows in panel (b) indicate outward and counterclockwise lateral movement of the TM2 helices. (c) Side view of the ion-permeation pathway in the IVM-induced desensitized state. The pore lumen is represented with blue dots. Side chains of pore-lining residues are colored with red or blue for negative and positive charged residues and green or white for hydrophilic and hydrophobic residues, respectively. (d) Pore profile comparisons of the α7nAChR TMD + ICD in the desensitized (purple) and resting (blue) states. The data were average from 15 structures in each state, and the dashed lines show the standard deviations. The narrow pore radii in the TMD (purple) at E238 and L248 changed to 2.20 ± 0.45 and 2.44 ± 0.28 Å in the desensitized state from 3.47 ± 1.19 and 1.53 ± 0.36 Å in the resting state. Source data are provided in a source data file.

In an IVM-induced desensitized state, both the TMD and ICD of α7nAChR show conformational changes relative to the structure in the resting state (Figure 3, Supporting Information Figure S9). IVM binding expands the inter-subunit gap near the extracellular end of the TMD from ∼7.3 Å in the apo α7 to ∼10.8 Å in the α7-IVM complex, measured between Cα atoms of A276 and I217 in two adjacent subunits (Supporting Information Figure S9). Consistent with previously observed conformational changes accompanying desensitization of α7nAChRs and other Cys-loop receptors,^11,12,31−33^ the upper half of the TM2 of α7-IVM displays an outward radial movement and a counterclockwise lateral rotation (Figure 3a,b), leading the radial (θ) and lateral (φ) tilting angles^34^ from θ = −1.0 and φ = −2.5° in the resting state (PDBID: 7RPM) to θ = 6.8° and φ = 0.4° in the desensitized state (PDBID: 8F4V). These structural changes result in a profile of the ion-permeation pathway that is nonconductive, but distinctly different from the resting state in that the narrowest construction is located closer to the ICD at E238 (2.20 ± 0.44 Å) with a second constriction (which is the main one for the resting state) at L248 (9′) (2.44 ± 0.28 Å) (Figure 3c,d). These constrictive radii are too small to pass a hydrated Na^+^ or Ca^2+^ ion.^35^ The overall pore profile of α7-IVM shares the features demonstrated in previously reported structures of α7nAChRs and other Cys-loop receptors in a desensitized state^11,12,31−33^ (Supporting Information Figure S10).

**Figure 4:**
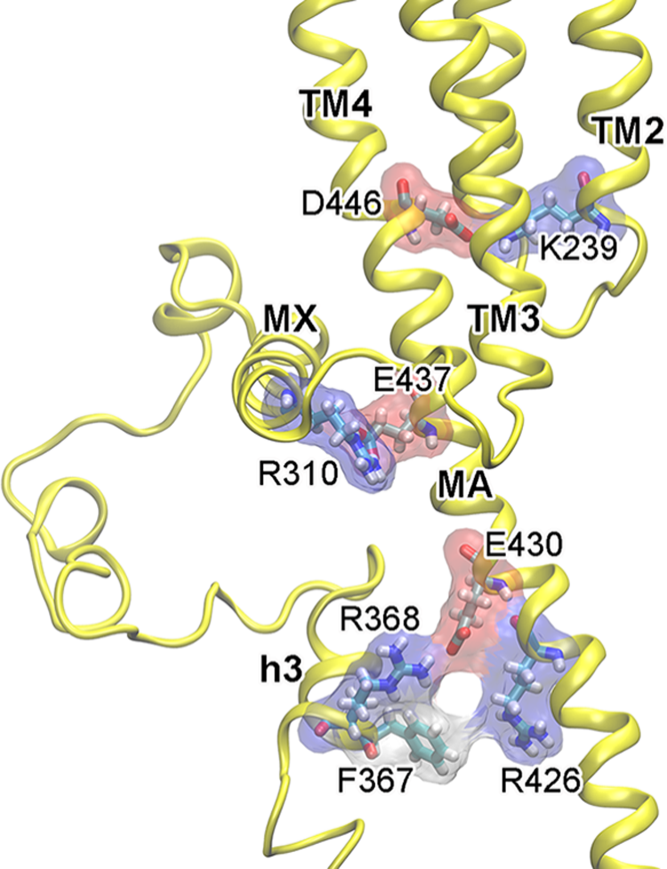
Representative interactions stabilizing ICD structures. Salt bridges found in the ICD of a subunit in the resting state remain in the IVM-induced desensitized state, but the contact between the F367 and R426 side chains is lost.

**Figure 5:**
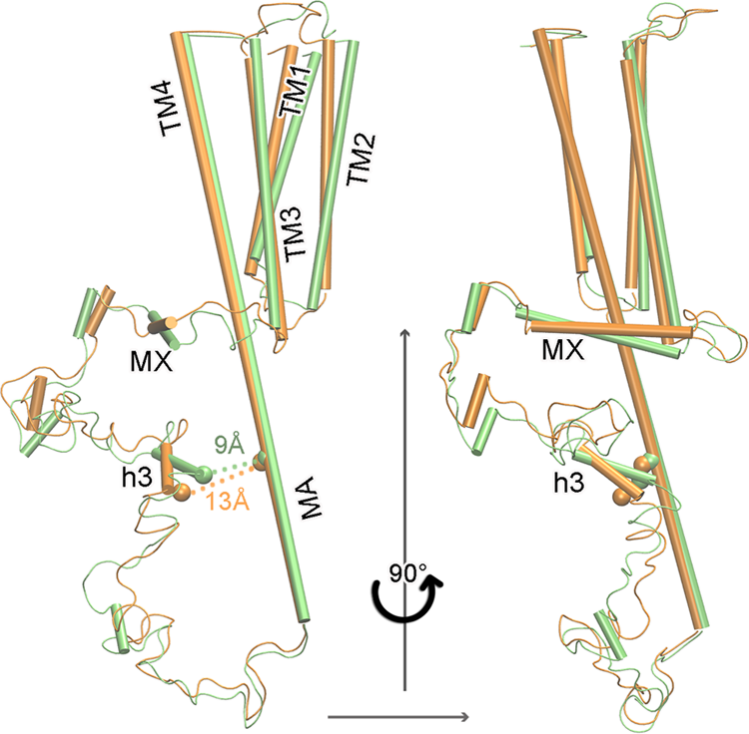
Structure comparison of the α7nAChR TMD+ICD in the IVM-induced desensitized state with the resting state. The aligned structures representing the resting (lime) and desensitized (orange) states show profound displacements not only in the TM1–TM3 but also in the ICD, including MX and h3 helices.

In the ICD, each subunit contains a flexible loop L (R322-D408) connecting MX (W308-L321) and MA (P409-C443) helices. The overall “B” shape folding of loop L observed in the resting structures^13^ is maintained in a desensitized state (Figure 3a). Salt bridges found in the resting state also exist in the desensitized structure (Figure 4). A K239-D446 salt bridge links movements of the TM2 and TM4 helices in different functional states,^11^ and D446 is critical for expressing functional α7nAChRs on the cell surface.^36^ Salt bridges R310-E437 and R368-E430 anchor the respective MX and h3 helices to the MA helix that stabilize ICD tertiary structures. Residue F367 on h3 is more distal from R426, and their Cα–Cα distance changed from ∼9 Å in the apo structures to ∼13 Å in the α7-IVM structures (Figure 5). This is largely due to a change of h3 tilting relative to the channel axis from ∼70° in the apo structure to ∼38° in the α7-IVM structure (Figure 5). The orientation of the MX helix is adjusted from ∼75 to 89°, making the MX helix almost perfectly perpendicular to the channel axis (Figure 5). Despite its uptilted appearance compared to the orientation in the apo structure (Figure 5), the MX helix does not move upward and outward as shown in the “open” channel structures of α7nAChR^11,12^ and the 5-HT_3A_ receptor.^32^

### MD Simulations of the α7-IVM Complex

**Figure 6:**
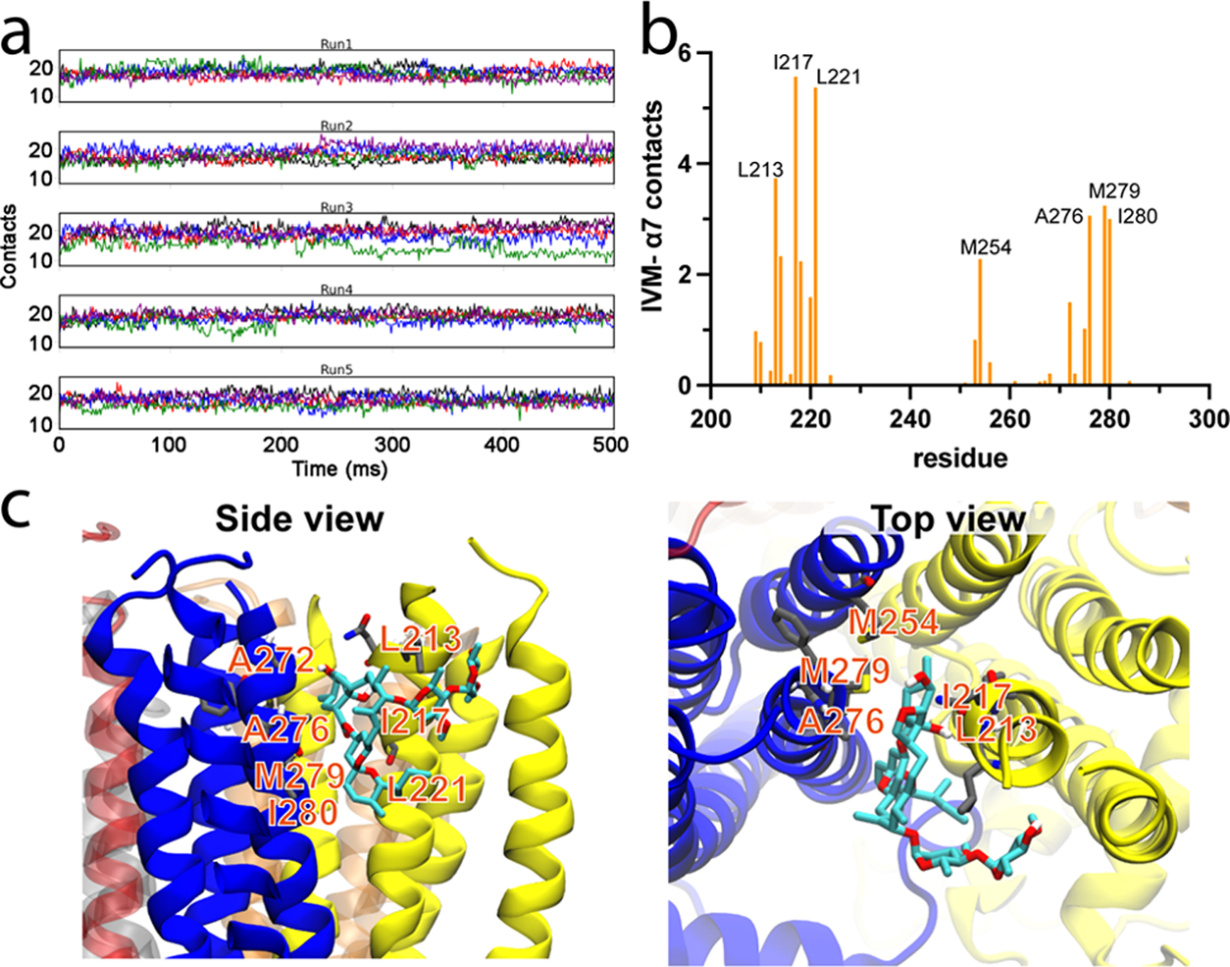
IVM interactions with α7nAChR TMD + ICD captured in MD simulations. (a) Overall IVM-α7 contacts during five replicating MD simulations in the POPC/POPA/Chol membrane system. A contact is counted if two atoms from IVM and α7 are within 3.5 Å. Each color represents one of five IVM molecules in the TMD + ICD pentamer. (b) Number of contacts of IVM to individual residues of α7nAChR in each binding interface in each frame of MD simulations in panel (a). (c) Molecular view of IVM interacting with α7 residues captured in MD simulations.

To assess the stability of IVM binding in the α7nAChR TMD+ICD, we performed multiple replicates of MD simulations on a representative NMR structure of the α7-IVM complex embedded into a homogeneous membrane of 1-palmitoyl-2-oleoyl phosphatidylcholine (POPC) or a heterogenous membrane of POPC, 1-palmitoyl-2-oleoyl phosphatidic acid (POPA), and cholesterol (Chol) lipid molecules in 3:1:1 ratio.^37,38^ During simulations, we observed no significant deviation of IVM from its initial binding site (Supporting Information Figure S11). IVM maintained stable hydrophobic interactions and hydrogen bonding with α7 residues in both membrane systems and all of the replicates (Figure 6, Supporting Information Figures S12 and S13). The overall contact patterns in both lipid systems are similar (Supporting Information Figure S14). The benzofuran ring wedges deep into the inter-subunit binding site, forming hydrogen bonds with main chain oxygen of L213 and side chain N214. It also interacts with hydrophobic residues P218, M254, A272, F275, A276, and M279 (Figure 6 and Supporting Information Figure S14). The spiroketal moiety faces toward the cytoplasm and interacts with I217, L221, A276, M279, and I280. The disaccharide moiety is mostly outside the binding cleft facing the extracellular side.

The new experimental data and models reported here provide structural illumination of IVM binding to the homo-pentameric human α7nAChR TMD + ICD. Despite certain variations in IVM-contacting residues, the NMR-identified IVM binding site at the interface of adjacent subunits in the α7nAChR TMD has a sequence motif with good overall similarity to the IVM sites found in GluCl and glycine receptors,^15−18^ and the IVM binding poses also exhibit similarities. In addition to the NMR-revealed insights into IVM binding, MD simulations corroborate stable IVM binding to the α7nAChR TMD site. The molecular details and recurring patterns of this binding provide interesting structural insight that should be possible to use for developing new α7nAChR modulators. The α7-IVM complex structures obtained from the current studies are consistent with functional measurements of desensitization of the α7nAChR TMD + ICD channel after prolonged IVM application. The structural changes between the resting and desensitization states are observed in not only the TMD but also the ICD. Since available full-length ICD structures are limited, the identified changes of the ICD in different functional states are particularly valuable for understanding intracellular signaling mediated by interactions between the ICD and various cytoplasmic proteins.

## Materials and Methods

### Protein Expression and Purification and Sample Preparations

The current studies used the same protein constructs and production protocols as used previously for structure determination of the resting-state TMD + ICD.^13^ Full-length human α7nAChR^39^ and TMD + ICD were expressed in *Escherichia coli* Rosetta 2(DE3) pLysS (Novagen)^39^ in LB broth or M9 minimal media at 15 °C for ∼16 or ∼72 h, respectively. As reported previously,^13,39^ proteins were purified with NiNTA resin (GE Healthcare), and the pentamer fraction was isolated by size exclusion chromatography (SEC) using a S200 10/300 column (GE Healthcare) equilibrated with 20 mM HEPES pH 7.4, 300 mM NaCl, 0.2% DPC, or 0.1% LDAO. Protein purity was confirmed by SDS-PAGE. MTSL labeling of unpaired single-cysteine full-length α7nAChR or the TMD + ICD α7nAChR was achieved by the protocol published previously.^40,41^ After removing the reducing agent DTT by PBS buffer exchange at pH 8, we added a ∼15- to 25-fold molar excess of the nitroxide spin label MTSL (Toronto Research Chemicals) to protein samples for ∼2 h at room temperature, followed by overnight incubation at 4 °C to ensure >90% labeling efficiency for NMR. For ESR, the desired 60–80% labeling efficiency was achieved by adjusting the MTSL amount and labeling time. Free MTSL was removed through dialysis and subsequent SEC. The TMD + ICD α7nAChR in LDAO micelles was directly used for NMR. A typical NMR sample contained 0.2–0.3 mM protein in ∼1% LDAO micelles, pH 4.7, and 25 mM NaCl with 5% D_2_O for deuterium lock. Full-length α7nAChR was reconstituted in liposomes for ESR, for which solubilized asolectin was added to purified protein in detergent at a 10-fold weight excess and gently agitated for 1 h before removing detergent using BioBeads. The liposomes were then collected by ultracentrifugation at 200,000*g* for 1 h and resuspended by sonication (FB11207 FisherScientific) in PBS pH 7.4 prepared in D_2_O. A typical ESR sample contained 0.1–0.2 mM protein in liposomes in phosphate-buffered saline pH 7.4 prepared in D_2_O. IVM (Sigma-Aldrich) was first dissolved in DMSO (Fisher Bioreagents) and then added to NMR samples to a desired concentration. The same quantity of DMSO was included in control samples without IVM to exclude a potential DMSO effect.

### Electrophysiology Measurements

TEVC measurements of *Xenopus* oocytes injected with purified α7nAChR TMD + ICD (Figure 1a) were performed as previously reported.^13^ Briefly, 5 ng of the purified α7nAChR TMD + ICD reconstituted in asolectin liposomes was injected into *Xenopus laevis* oocytes (stages 5–6). One to two days after the injection, channel function was measured in a 20 μL oocyte recording chamber (Automate Scientific) perfused at 2.4 mL/min and clamped at −60 mV with an OC-725C amplifier (Warner Instruments). Recording solutions contained 96 mM NaCl, 2 mM KCl, 1.8 mM CaCl_2_, 1 mM MgCl_2_, and 5 mM HEPES at pH 7.0. Data were collected using Clampex 10.6 (Molecular Devices) and processed with Clampfit 10.6 (Molecular Devices). The oocytes used in this study were kindly provided by Dr. Thomas Kleyman’s lab after harvesting from commercial female *X. laevis* (Xenopus 1, MI). All animal experimental procedures were approved by the Institutional Animal Care and Use Committee (IACUC) of the University of Pittsburgh.

### NMR Spectroscopy

NMR spectra were acquired at 318 K on a Bruker Avance 800 MHz spectrometer equipped with a triple-resonance inverse-detection TCI cryoprobe (Bruker Instruments). TopSpin 3.2 (Bruker) was used for acquisition, NMRPipe 3.19^42^ for processing, and NMRFAM-SPARKY 1.470^43^ powered by Sparky 3.190^44^ for NMR spectral analysis. A list of NMR experiments and the most relevant data acquisition parameters are summarized in Supporting Information Table S4. A relaxation delay of 1 s was used in all NMR spectra, except 1D and 2D STD NMR experiments. The ^1^H chemical shifts were referenced to the DSS resonance at 0 ppm, and the ^15^N and ^13^C chemical shifts were referenced indirectly. STD^25,27,45^ (Figures 2 and S3 and S4) and chemical shift perturbation NMR experiments (Figures 1b and S1) were used to determine the IVM binding site in the α7nAChR TMD + ICD. STD spectra were collected in an interleaved fashion with off- and on-resonance saturation of selected IVM ^1^H peaks for 2 s saturation and a recycle time of 3 s. The off-resonance frequency was set at 20 ppm, which is far away from ^1^H frequencies of the protein and IVM. The on-resonance IVM frequencies were selected as indicated in individual spectra. The selective saturation was achieved using an IBURP2 pulse train (50 ms Gaus1.1000-shaped with an inter-pulse delay of 4 μs). The 1D ^1^H STD spectra were recorded with a spectral window of 16 ppm and 16,384 data points. For IVM ^1^H chemical shift assignment, 2D NOESY and TOCSY spectra were recorded with mixing times of 250 and 60 ms, respectively, at 318 K on a Bruker Avance 600 MHz spectrometer equipped with a triple-resonance inverse-detection TCI cryoprobe (Bruker Instruments). Spectral width of 10 × 10 ppm and 1024 × 256 data points were used.

2D ^1^H-^15^N TROSY–HSQC saturation transfer spectra have windows of 13 × 23 ppm and 2048 × 160 data points in the ^1^H and ^15^N dimension, respectively. Decreases in the α7 TMD-ICD cross-peak intensity upon saturation of the IVM ^1^H peaks were analyzed and used to determine direct interactions between the α7 residues and IVM. The chemical shift perturbation NMR experiments were performed with ^1^H-^15^N TROSY–HSQC of the α7nAChR TMD + ICD in the presence of 0, 30, 100, 250, and 500 μM IVM using spectral windows of 13 × 23 ppm and 2048 × 160 data points in the ^1^H and ^15^N dimension, respectively. 3D TROSY–HNCO NMR spectra of the α7nAChR TMD + ICD in the absence and presence of IVM were also collected to aid in determination of IVM binding in the TMD + ICD (Figure S2). Spectral width of 12 × 23 × 12 ppm and 1024 × 48 × 56 data points were set in the ^1^H_N_, ^15^N, and ^13^C dimensions, respectively. Distance restraints for structure calculations of the α7nAChR TMD + ICD in an IVM-induced desensitized state were obtained from 2D PRE NMR experiments as used previously for determination of the α7nAChR TMD + ICD in the resting state.^13^^1^H-^15^N TROSY–HSQC spectra in the paramagnetic (I) and diamagnetic (I_0_) conditions were acquired for each of eight MTSL-labeled single-cysteine TMD + ICD constructs in the presence of ∼200 μM IVM (Figures S5 and S6). Spectral windows of 13 × 23 ppm and 2048 × 176 data points were used in the ^1^H_N_ and ^15^N dimensions, respectively. The diamagnetic condition was introduced by ascorbic acid (2–2.5 mM). The same spectra, but without IVM, were also collected as a control of the PRE data for the α7 TMD + ICD in the resting state. Distance restraints were derived using the Solomon and Bloembergen equation^46,47^ and used along with other structural restraints for structure calculations.^13^

### ESR Spectroscopy

Four pulse DEER experiments were performed on full-length α7nAChR using a Bruker ElexSys E580 Q-band CW/FT spectrometer equipped with an ER 5106-QT2 resonator or a Bruker ElexSys E680 CW/FT X-band spectrometer equipped with a Bruker EN4118X-MD4 resonator (Figure S7). Each sample contained a spin concentration of 60–160 μM and was prepared using deuterated water and glycerol (20% v/v) as a cryoprotectant for increasing relaxation times and flash frozen in 2 × 3 mm^2^ or 3 × 4 mm^2^ tubes for Q-band or X-band, respectively. The DEER pulse sequence comprises [(π/2)_ν1_ – τ_1_ - (π)_ν1_ – *t* + d*t* – (π)_ν2_ – τ_2_ - (π)_ν1_ – τ_2_ – echo].^48^ The pump frequency (ν2) was set 70 MHz up-field from the observer frequency (ν1). The pulse lengths of (π)_ν1_ and (π)_ν2_, pump pulse step size (d*t*), and the number of data points (*n*) were optimized for individual samples, with τ_1_ of 400 ns and τ_2_ being slightly larger than n*d*t*. The time domain DEER signal was analyzed with DD.^49^

### Modeling of the IVM-α7 TMD + ICD Complex

Using HADDOCK2.4,^28,29^ we took the STD NMR results in combination with chemical shift perturbation data as experimental restraints to guide IVM docking to an ensemble structure of the α7nAChR TMD + ICD in a desensitized state generated from Rosetta (see below). The IVM structure was extracted from a crystal structure of α3GlyR-IVM complex^16^ (PDBID: 5VDH). The standard HADDOCK protocols were followed with the modification that (i) the number of trials for rigid body minimization was increased from 5 to 10 and (ii) as a solvent, water was replaced by DMSO that better mimics the environment for the TMD. Finally, the 15 structures with the best HADDOCK scores and lowest restraints violation energy were chosen for PDB deposition (PDBID: 8F4V).

### Comparative Modeling in Rosetta

The protocol used for the resting-state TMD + ICD structures^13^ was applied for the desensitized structures with new experimental structural restraints. The comparative modeling protocol (RosettaCM)^50^ in Rosetta 3.7^51^ with the talaris2014 energy function^52^ was used for structure calculations. Iterative structural calculations were performed on Open Science Grid.^53^ Experimental structural restraints included hydrogen bonds, NMR NOE and PRE, and ESR DEER distance restraints (Table S2) as well as the 3- and 9-residue structural fragment library previously generated^13^ using CS-Rosetta^54^ on the Robetta server^55^ with input chemical shifts, RDC, and NOE data. Fivefold symmetry was applied in structural calculations. An initial desensitized structural model was generated using the TMD of the α3GlyR-IVM crystal structure^16^ along with the ICD in a representative structure of the resting-state TMD + ICD^13^ as a template. With specified experimental restraints, an iterative folding protocol was run four times. Each iteration generated 1000 structures, which were ranked later by a total score *S*_total_ that includes the standard weighted physics-based (*S*_physics_) and knowledge-based (*S*_knowledge_) potentials from the talaris2014 energy function^52^ and the experimental restraint potentials. The top 100 structures were clustered with a 3 Å RMSD cutoff^56^ using Matlab 2020b (Mathworks), and the top ranked structures from each cluster were input as new template structures for the next iteration of folding calculations. At the end of the 4th run, the 50 lowest-score structures were refined with Rosetta FastRelax^57^ followed by Chiron^58^ to minimize steric clashes and Phenix 1.19^59^ for geometry optimization. The structures were validated by the *Q*-factor^60^ (analogous to the crystallographic *R*-factor) as reported previously^13^ with 56 PRE restraints that were not used in structure calculations. These structures were used for NMR restraint-guided IVM docking using HADDOCK.^28,29^ The final selection of 15 structures was based on the best HADDOCK scores and lowest restraints violation energy. The quality of the final structures was evaluated using MolProbity^61^ and Phenix^59^ 1.19 (Table S3). VMD^62^ was used for structure rendering, visualization, and analysis. Pore profiles were calculated using the HOLE program.^63^

### MD Simulations

Two types of simulation systems were prepared by embedding a representative IVM-bound NMR structure into (i) a homogeneous membrane of 1-palmitoyl-2-oleoyl phosphatidylcholine (POPC) lipids and (ii) a heterogenous membrane of POPC, 1-palmitoyl-2-oleoyl phosphatidic acid (POPA), and cholesterol (Chol) lipids in 3:1:1 ratio.^37,38^ The first system is to represent a model membrane, and the second is to a realistic one.^36^ The systems were built using the Membrane Builder module of CHARMM-GUI.^64^ Each system was solvated with TIP3P water^65^ and neutralized in 0.15 M KCl to generate systems, each containing ∼200,000 atoms with dimensions of 120 × 120 × 150 Å^3^. To allow for better sampling, five independent replicas of each system were built by randomly configuring initial lipid placement around the protein–ligand system.^166^ Following the default CHARMM-GUI settings, each system was energy-minimized and then relaxed in simulations at constant pressure (1 bar) and temperature (300 K) for 25 ns, during which the position restraints on the protein were gradually released. For production runs, all restraints were released except Cα atoms of the ICD and pore-facing TMD helix that were restrained with a mild force constant of 20 KJ mol^–1^nm^–2^. These restraints were applied to maintain the highly dynamic part of the protein in the positions determined experimentally. MD simulations were performed using GROMACS-2022^66^ utilizing CHARMM36m^67^ force field parameters for proteins and lipids, respectively. The force field parameters for IVM were generated using the CHARMM General Force Field (CGenFF).^68−70^ Bonded and short-range nonbonded interactions were calculated every 2 fs, and periodic boundary conditions were employed in all three dimensions. The particle mesh Ewald (PME) method^71^ was used to calculate long-range electrostatic interactions with a grid density of 0.1 nm^–3^. A force-based smoothing function was employed for pairwise nonbonded interactions at 1 nm with a cutoff of 1.2 nm. Pairs of atoms whose interactions were evaluated were searched and updated every 20 steps. A cutoff (1.2 nm) slightly longer than the nonbonded cutoff was applied to search for the interacting atom pairs. Constant pressure was maintained at 1 bar using the Parrinello–Rahman algorithm.^72^ Temperature coupling was kept at 300 K with the v-rescale algorithm.^73^

## Data Availability

The atomic coordinates and structural restraints for 15 structures of the α7nAChR TMD + ICD in complex with IVM have been deposited in the Protein Data Bank with the accession code 8F4V. The chemical shift values have been deposited in the Biological Magnetic Resonance Data Bank (BMRB), accession number 31058 [https://dx.doi.org/10.13018/BMR31058]. MD simulation trajectories were deposited into Zenodo (https://zenodo.org/record/7415043). Other data that support the findings of this study are available upon reasonable request to the corresponding author.

## Supporting Information Available

Results showing NMR chemical shift changes in the absence and presence of ivermectin (Figure S1), IVM-induced chemical shift changes observed in 3D experiments (Figure S2), selective saturation of ^1^H NMR signals of ivermectin (Figure S3), overlay of the 2D saturation transfer spectra of the α7nAChR TMD + ICD (Figure S4), PRE NMR for structure determination of the α7nAChR TMD + ICD in an IVM-induced desensitized state (Figure S5), quantified changes of normalized PRE (I*/*I_0_) from different MTSL-labeling sites based on their corresponding NMR spectra (Figure S6), representative DEER ESR data for quaternary structure restraints of α7nAChR in a desensitized state (Figure 7), a stereo image of structures of the desensitized α7nAChR TMD + ICD (Figure S8), IVM binding expands the inter-subunit gap near the extracellular end of the TMD (Figure S9), pore profile comparisons of the desensitized α7nAChR TMD + ICD with previously published desensitized structures (Figure S10), RMSD of IVM heavy atoms over the course of MD simulations in two different membrane systems (Figure S11), IVM-protein contacts over the course of MD simulations in two membrane systems (Figure S12), hydrogen bonds between IVM and α7nAChR over the course of MD simulations in two membrane systems (Figure S13), and average contacts of different IVM moieties to individual residues of α7nAChR in each frame of MD simulations (Figure S14). Sequence alignment of IVM binding sites (Table S1), NMR and ESR experimental restraints for α7nAChR structure calculations (Table S2), statistics for the 15 refined α7nAChR structures in a desensitized state (Table S3), and NMR samples, experiments, and most relevant acquisition parameters (Table S4) (PDF)
Source data underlying Figures 3c,d and 6b and Figures S1, S6, S7, S10, and S14 (XLSX)
