# Supplementary material for: Structural Elucidation of Ivermectin Binding to α7nAChR and the Induced Channel Desensitization
Source: ACS Chem Neurosci. 2023 Feb 23;14(6):1156–65. doi: 10.1021/acschemneuro.2c00783 (PMC10020961; doi:10.1021/acschemneuro.2c00783)
Supplement: Supplementary file 1 — cn2c00783_si_001.pdf [file cn2c00783_si_001.pdf]

Supporting Information for Publication

Structural Elucidation of Ivermectin Binding to α7nAChR and the Induced Channel Desensitization

Vasyl Bondarenko[1], Qiang Chen[1], Kevin Singewald[2], Nandan Haloi[3], Tommy S. Tillman[1], Rebecca J. Howard[3], Erik Lindahl[3], Yan Xu[1,4,5,6], Pei Tang[1,4,7*]

[1]Department of Anesthesiology and Perioperative Medicine, University of Pittsburgh, Pittsburgh, PA 15260, USA
[2]Department of Chemistry, University of Pittsburgh, Pittsburgh, PA 15260, USA
[3]Department of Biochemistry and Biophysics, Science for Life Laboratory, Stockholm University, Solna, Sweden; Department of Applied Physics, Swedish e-Science Research Center, KTH Royal Institute of Technology, Solna, Sweden
[4]Department of Pharmacology and Chemical Biology, University of Pittsburgh, Pittsburgh, PA 15260, USA
[5]Department of Structural Biology, University of Pittsburgh, Pittsburgh, PA 15260, USA
[6]Department of Physics and Astronomy, University of Pittsburgh, Pittsburgh, PA 15260, USA
[7]Department of Computational and Systems Biology, University of Pittsburgh, Pittsburgh, PA 15260, USA

[*]Correspondence and requests for materials should be addressed to P.T. (ptang@pitt.edu)

There are 15 Supporting Figures and four Supporting Tables

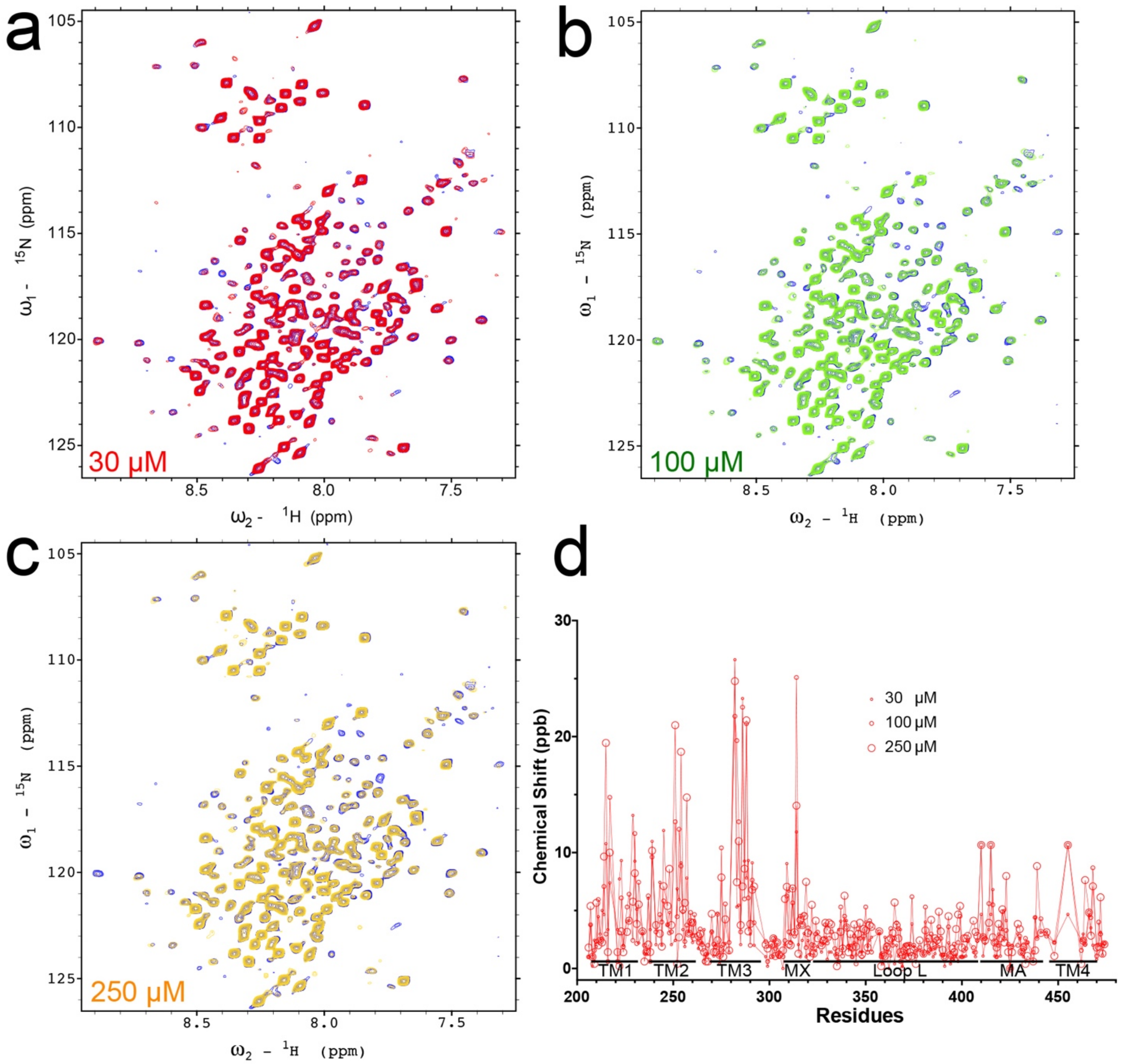

Fig. S1. NMR chemical shift changes in the absence and presence of ivermectin (IVM). Overlay of representative ^{1}H-^{15}N TROSY-HSQC NMR spectra of α7nAChR TMD+ICD in LDAO micelles in the absence (blue) and presence of **(a)** 30 μM (red), **(b)** 100 μM (green), and **(c)** 250 μM (yellow) of IVM. The spectra were collected on an 18.8-tesla NMR spectrometer at 45ºC. **(d)** Chemical shift changes ($\Delta\delta_{HN} = [\Delta\delta_H^2 + \Delta\delta_N^2/25]^{1/2}$) of the TMD+ICD from the resting to a desensitized state induced by 30, 100, 250 μM IVM (shown by red spheres in small, medium, and large size, respectively). The data used in (d) were from **(a)**, **(b)**, and **(c)**. Source data are provided as a Source data file.

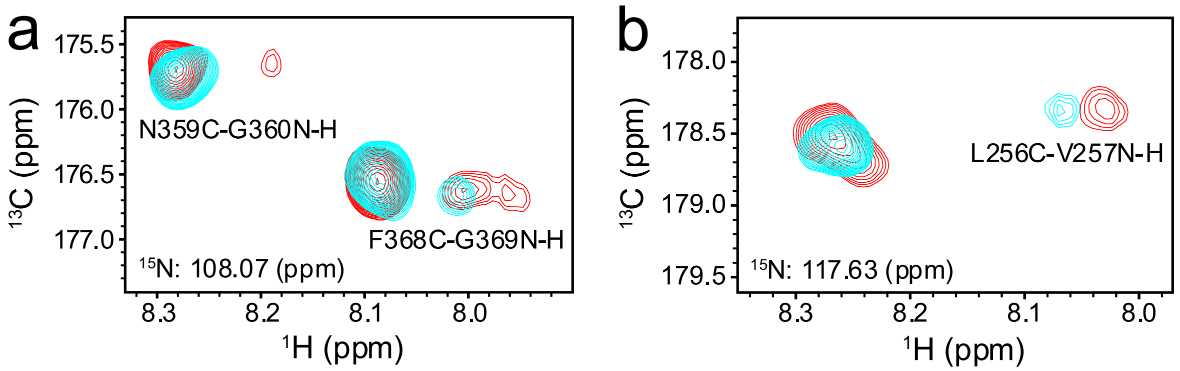

Fig. S2. IVM-induced chemical shift changes observed in 3D experiments. Overlay of representative zoom-in regions of 3D TROSY- HNCO NMR spectra of α7nAChR TMD+ICD in LDAO micelles in the absence (cyan) and presence (red) of 30 μM IVM. **(a)** Upon binding IVM, both G360 and G369 in the ICD show a chemical shift change. The second peaks may result from a slow chemical exchange. (**b**) V257 in the TM2 shows a chemical shift change after IVM binding. The spectra were collected on an 18.8-tesla NMR spectrometer at 45ºC.

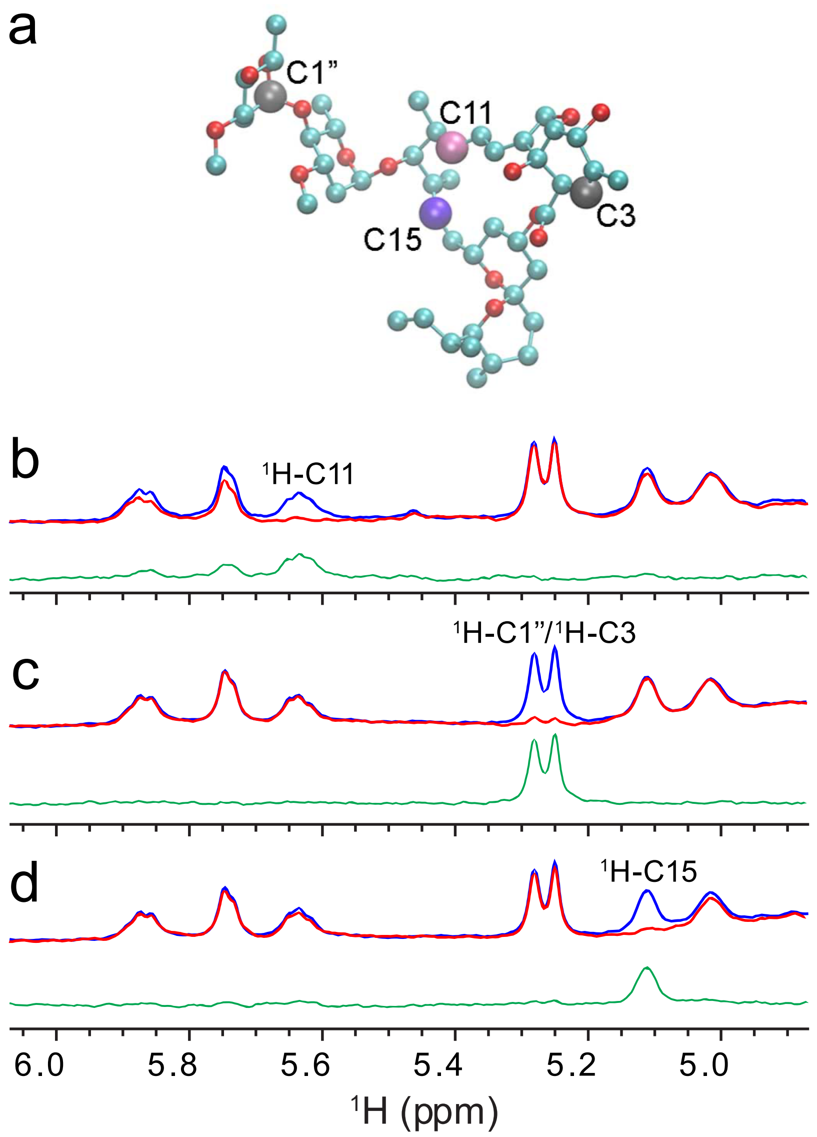

Fig. S3. Selective saturation of ^{1}H NMR signals of ivermectin (IVM). (a) Molecular structure of IVM labeled with carbon atoms[1] whose associating ^{1}H signals were selected for saturation in 1D STD NMR experiments. **(b)-(d)** Zoom-in regions of the on- (red) and off-resonance (blue) saturation spectra of IVM (0.5 mM) in the presence of α7nAChR TMD+ICD and LDAO micelles. Saturation of selected IVM peaks reached >90%. The difference spectra are shown below in green. ^{1}H peaks of IVM were assigned with 2D NOESY and TOCSY NMR spectra. The spectra were collected on an 18.8-tesla NMR spectrometer at 45ºC.

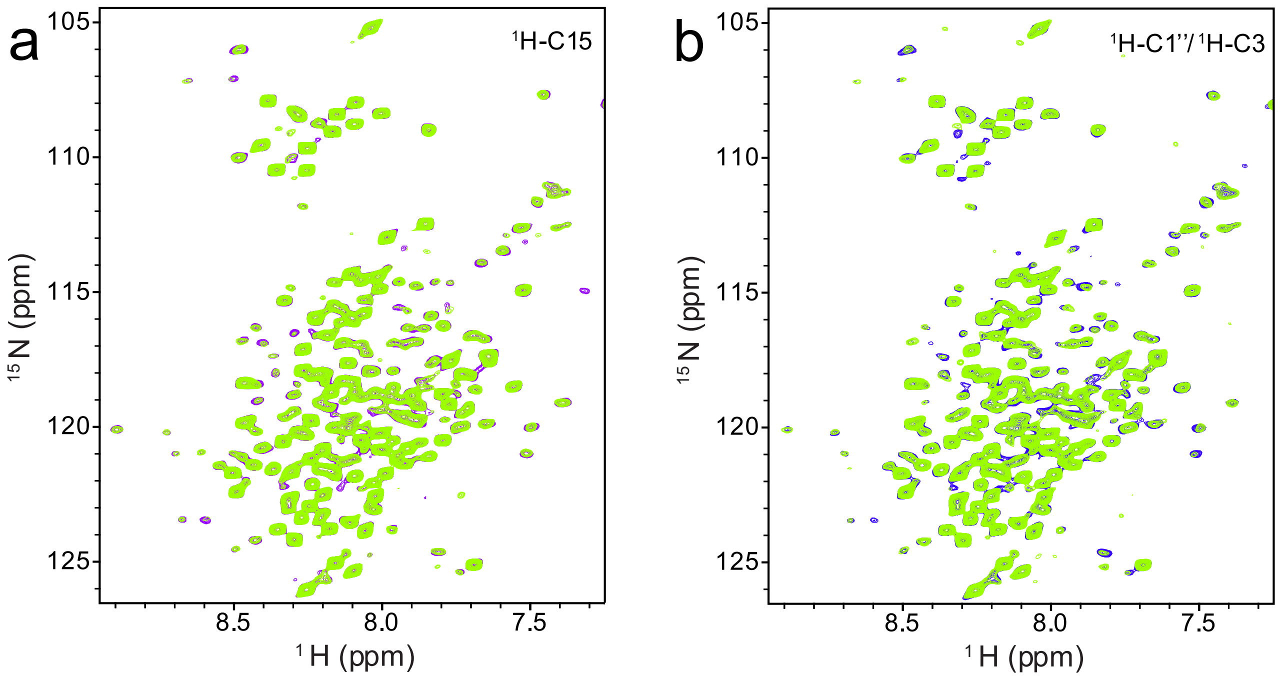

Fig. S4. Overlay of the 2D saturation transfer spectra of the α7nAChR TMD+ICD in LDAO micelles in the presence of 0.5 mM IVM without (blue) and with (green) ^{1}H resonance saturation at two different ^{1}H positions of IVM (up right corner). α7 residues in proximity to the saturated ^{1}H show intensity changes due to cross relaxation. The spectra were collected on an 18.8-tesla NMR spectrometer at 45ºC.

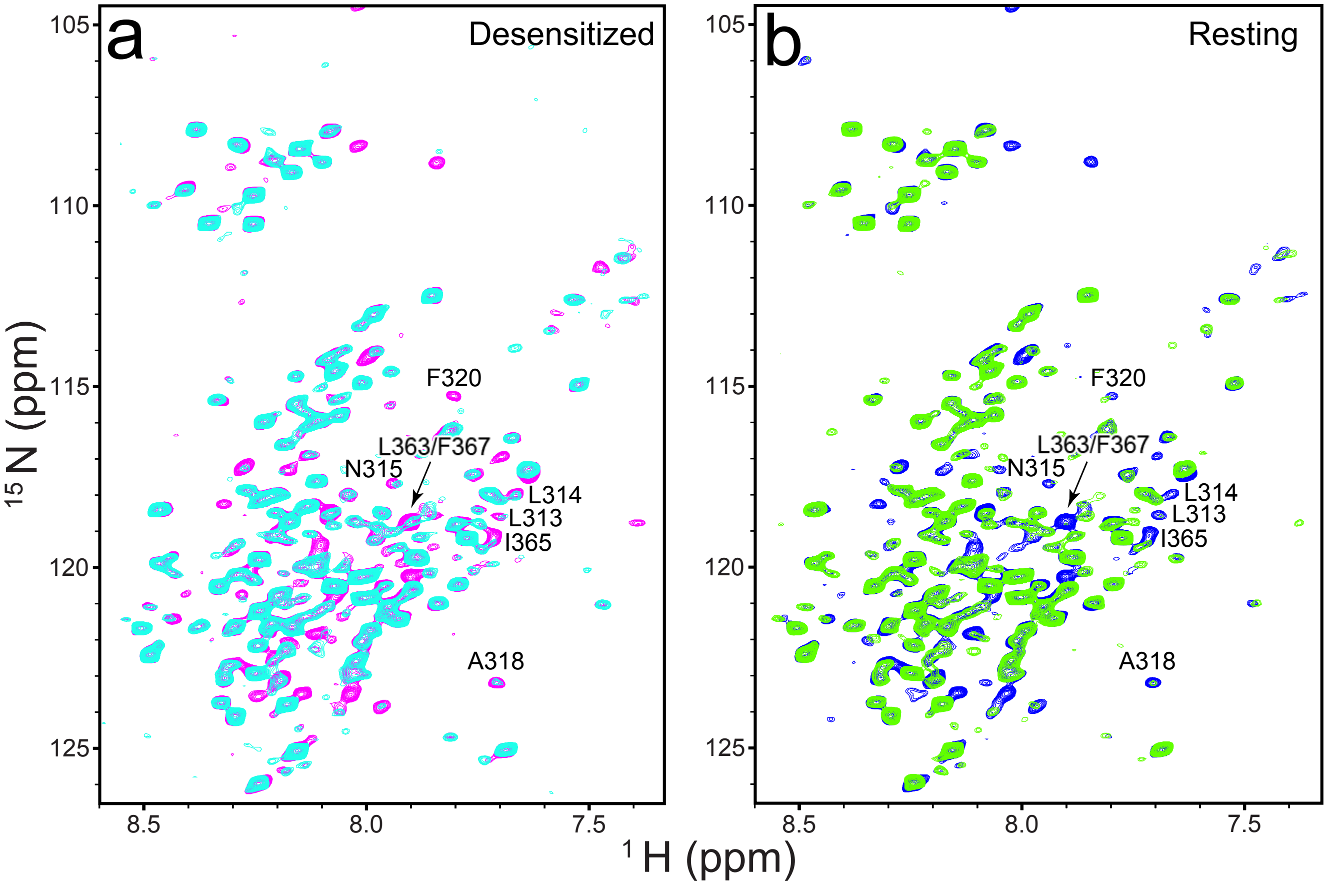

Fig. S5. **Paramagnetic Relaxation Enhancement (PRE) NMR for structure determination of the α7nAChR TMD+ICD in an IVM-induced desensitized state**. Representative ^{1}H-^{15}N TROSY-HSQC PRE NMR spectra of a single-cysteine α7nAChR TMD+ICD construct labeled with the paramagnetic MTSL at residue S350C in **(a)** a desensitized state induced by binding IVM (200 μM) and **(b)** the resting state without IVM otherwise identical protein sample in (a). Note peak intensity changes of certain residues in the paramagnetic condition (**a**-cyan; **b**-green) and diamagnetic condition (**a**-magenta; **b**-blue) induced by the reducing agent ascorbic acid. Moreover, note the labeled residues showing different PREs in the (a) desensitized and (b) resting states that reflect conformation changes in the regions where these residues are located. Quantified changes of normalized PRE (I/I_0) based on NMR spectra as shown in (a) and (b) are presented in **Fig. S6** (next page).

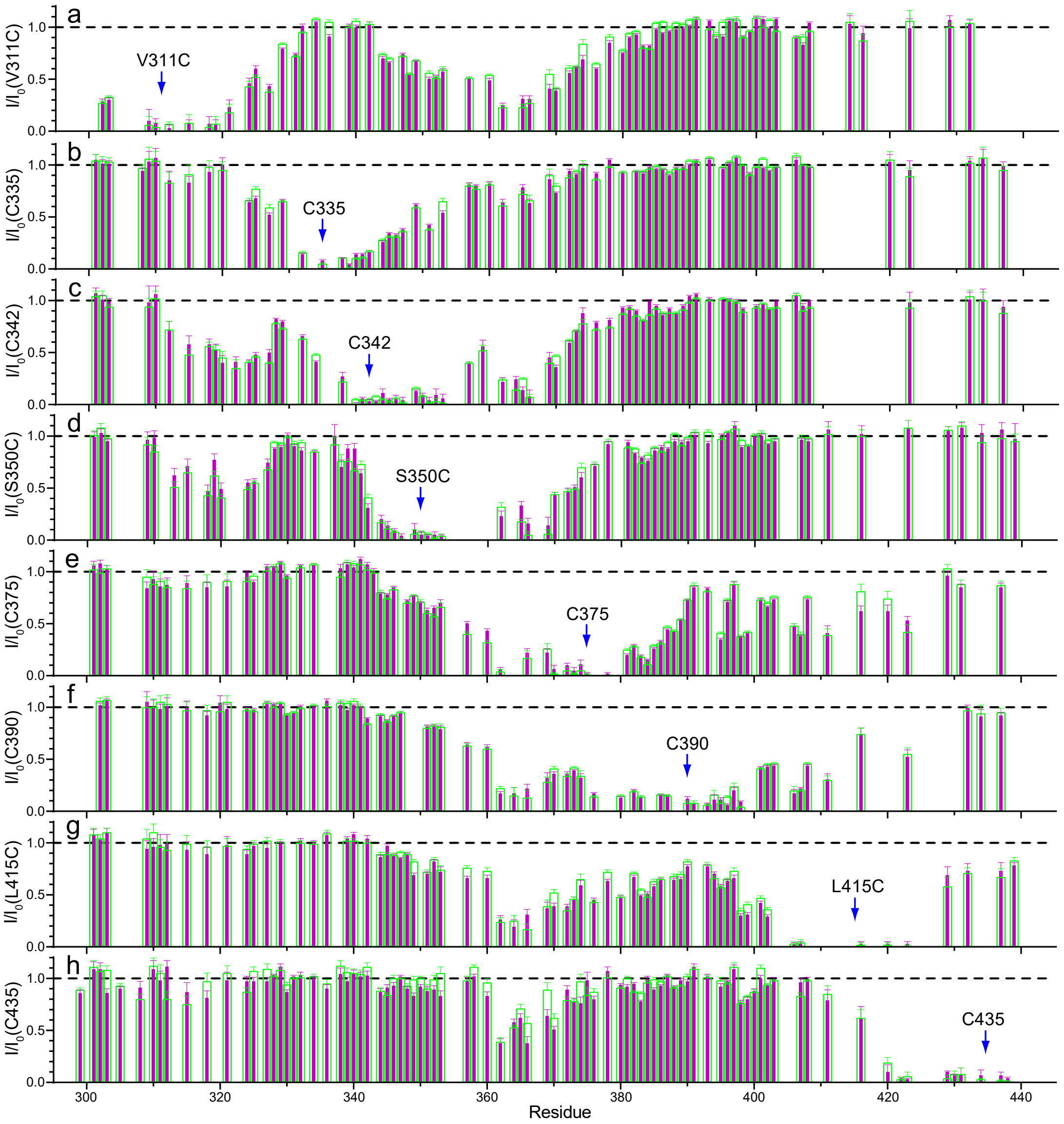

Fig. S6. **Quantified changes of normalized PRE (I/I_0) from different MTSL-labeling sites based on their corresponding NMR spectra** as shown in Fig. S5. A blue arrow with the label in each plot shows a location of individual paramagnetic MTSL-labeled residues, which include (**a**) V311C, (**b**) C335, (**c**) C342, (**d**) S350C, (**e**) C375, (**f**) C390, (**g**) L415C, (**h**) C435. I and I_0 represent NMR cross-peak intensities in the paramagnetic and diamagnetic states, respectively. $I/I_0 = 0$ or 1 (marked with dash lines) represents the maximum or no PRE. Residues severely overlapped with other residues in the NMR spectra are excluded from the plots. Error bars represent uncertainties derived from signal to noise of individual cross peaks for each independent experiment using the equation:

$$\Delta\left(\frac{I}{I_0}\right) = \frac{I}{I_0}\sqrt{\left(\frac{\Delta I}{I}\right)^2 + \left(\frac{\Delta I_0}{I_0}\right)^2}$$

where ΔI and ΔI_0 are the noise levels in the paramagnetic and diamagnetic NMR spectra,[2] respectively. Source data are provided as a Source data file.

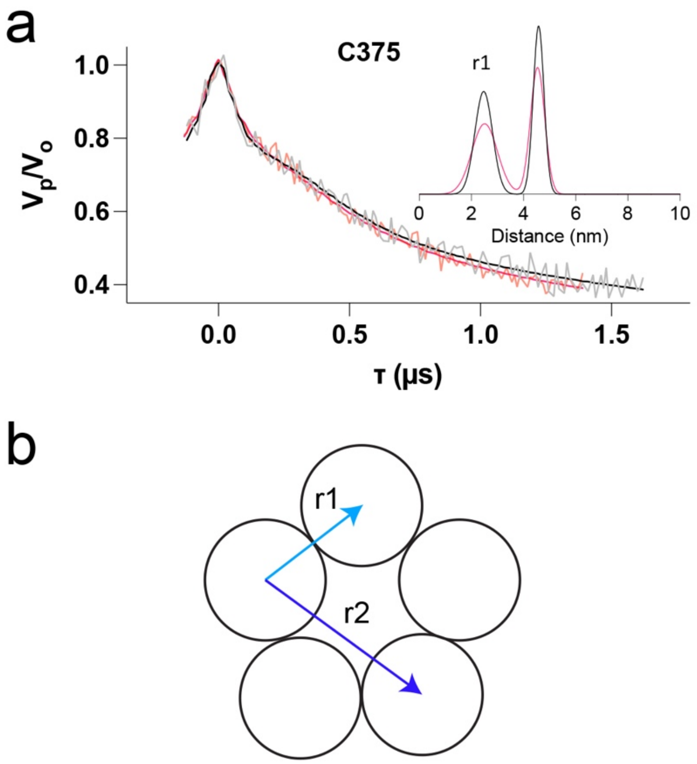

Fig. S7. **Representative DEER ESR data for quaternary structure restraints of α7nAChR in a desensitized state. (a)** X-band DEER of time domain signals with best fits of full-length α7nAChR C375 in asolectin liposomes in the presence (red) or absence (black) of 0.5 mM nicotine**.** Insets show the resulting adjacent (r1) and across (r2) distance distributions obtained from the DD[3], which was chosen as it can fit the DEER ESR data without the need for background subtraction and prevent artifacts and biases due to manual background subtraction. A two Gaussian model was found to be most optimal for fitting distances.[4] The resulting distance distributions were r1 = 2.51 ± 0.22 nm and r2 = 4.54 ± 0.47 nm in the presence of 0.5 mM nicotine; r1 = 2.47 ± 0.16 nm and r2 = 4.58 ± 0.32 nm in the absence of nicotine. The average distance and error of the two Gaussians serve as input parameters for Rosetta calculations. (**b**) r1 and r2 distances marked in the top view of a cartoon α7nAChR pentamer. DEER parameters were $(\pi)_{v1}$ = 24 ns, $(\pi)_{v2}$, = 12 ns, dt = 14 ns, n = 4410, and temperature = 80 K in the absence of nicotine and $(\pi)_{v1}$ = 32 ns, $(\pi)_{v2}$ = 16 ns, dt = 12 ns, n = 5312 and temperature = 80 K in the presence of nicotine. Distances are reported as mean ± 95% confidence interval. Source data are provided in a Source data file.

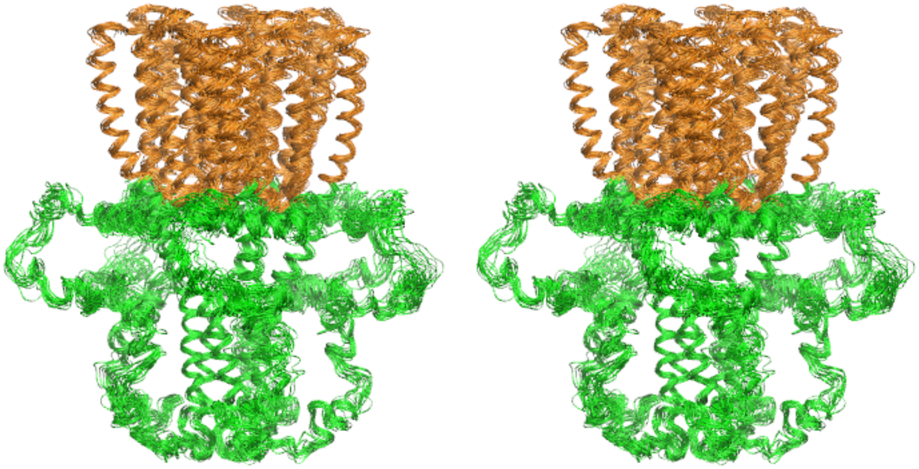

Fig. S8. A stereo image of structures of the desensitized α7nAChR TMD+ICD. The superimposed backbone traces are from the 15 lowest energy structures of the desensitized α7nAChR TMD (orange) + ICD (green). Their atomic coordinates and structural restraints have been deposited to the Protein Data Bank with accession code 8F4V.

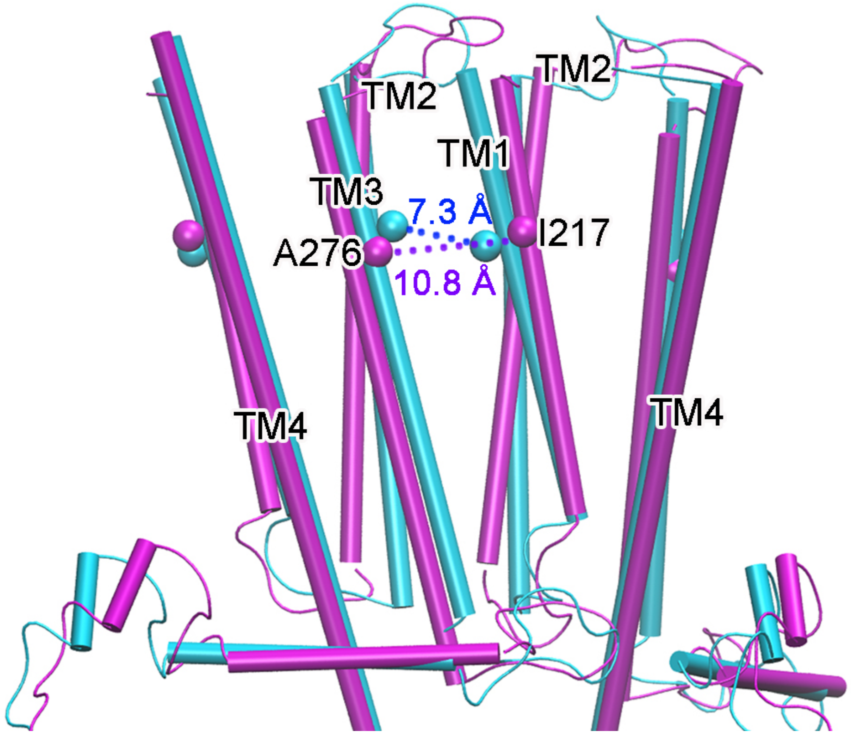

Fig. S9. IVM binding expands the inter-subunit gap near the extracellular end of the TMD. The **α7nAChR** TMD+ICD in the resting state (cyan, PDBID: 7RPM)[4] and the IVM-bound desensitized state (magenta, PDBID: 8F4V) are aligned. The gap between two adjacent subunits changed from ~7 Å in the resting state to ~11 Å in the IVM-bound desensitized state. The distances of the gap were measured by Cα atoms of the TM3 A276 in the principal subunit and the TM1 I217 in the complimentary subunit.

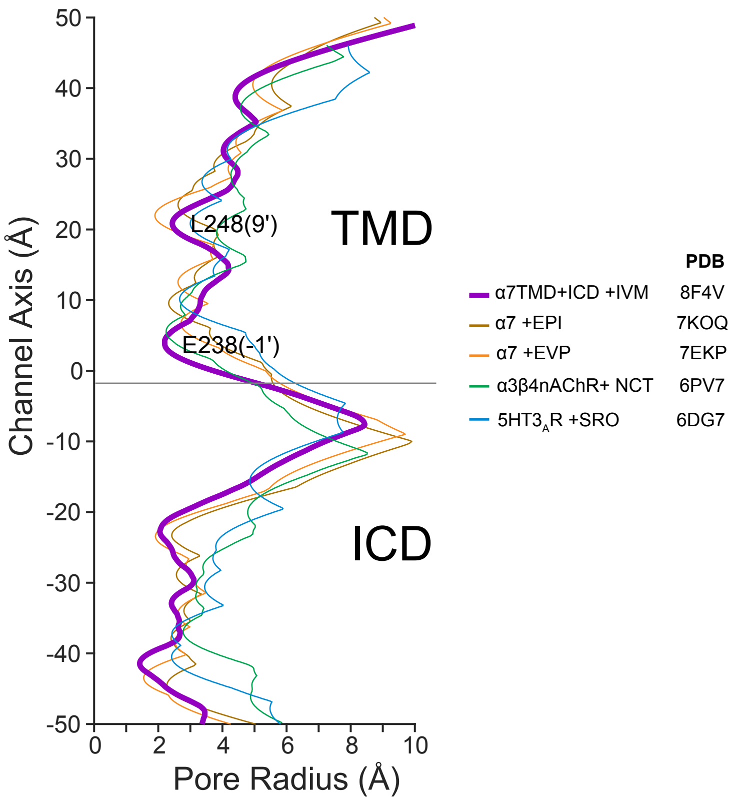

Fig. S10. Pore profile comparisons of the desensitized α7nAChR TMD+ICD with previously published desensitized structures. The averaged pore profile from 15 structures of the α7nAChR TMD+ICD (purple, PDBID: 8F4V) is compared with the pore profiles of desensitized channels, including epibatidine-bound α7nAChR (α7+EPI, brown, PDBID: 7KOQ),[5] EVP-6124-bound α7nAChR (α7+EVP orange, PDBID: 7EKP),[6] nicotine-bound α3β4nAChR (green, PDBID: 6PV7),[7] and serotonin-bound $5HT3_AR$, state 1 (cyan, PDBID: 6DG7).[8] The pore radius is calculated by HOLE[9] and plotted as a function of distance along the pore axis. The transmembrane domain (TMD) and intracellular domain (ICD) are marked. The pore profile of the newly determined structures of the desensitized α7nAChR TMD+ICD matches reasonably well with the pore profiles of the two α7nAChRs (brown and orange). Source data are provided as a Source Data file.

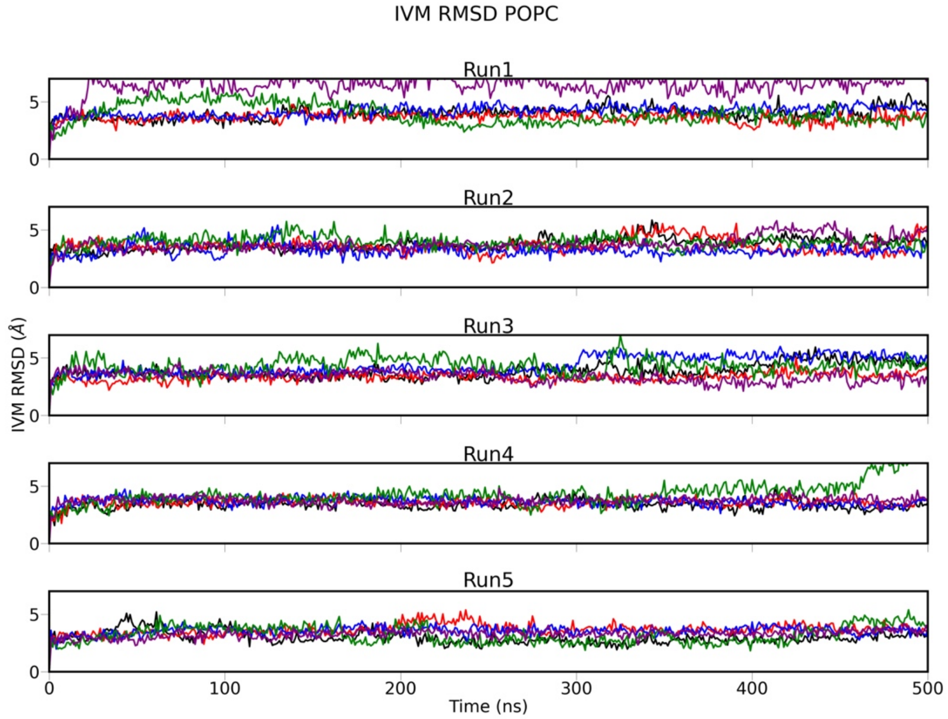

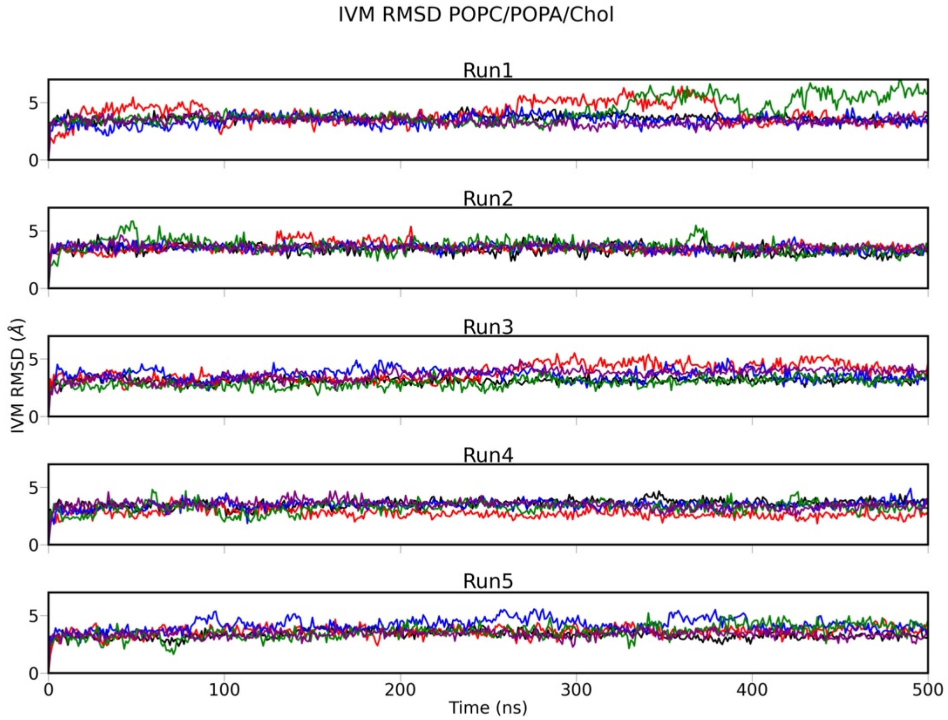

Fig. S11. Root means squared deviations (RMSD) of IVM heavy atoms over the course of MD simulations in two different membrane systems, POPC (top) and POPC/POPA/Chol (bottom). Each color represents one of five individual IVM molecules in the pentameric α7nAChR TMD+ICD.

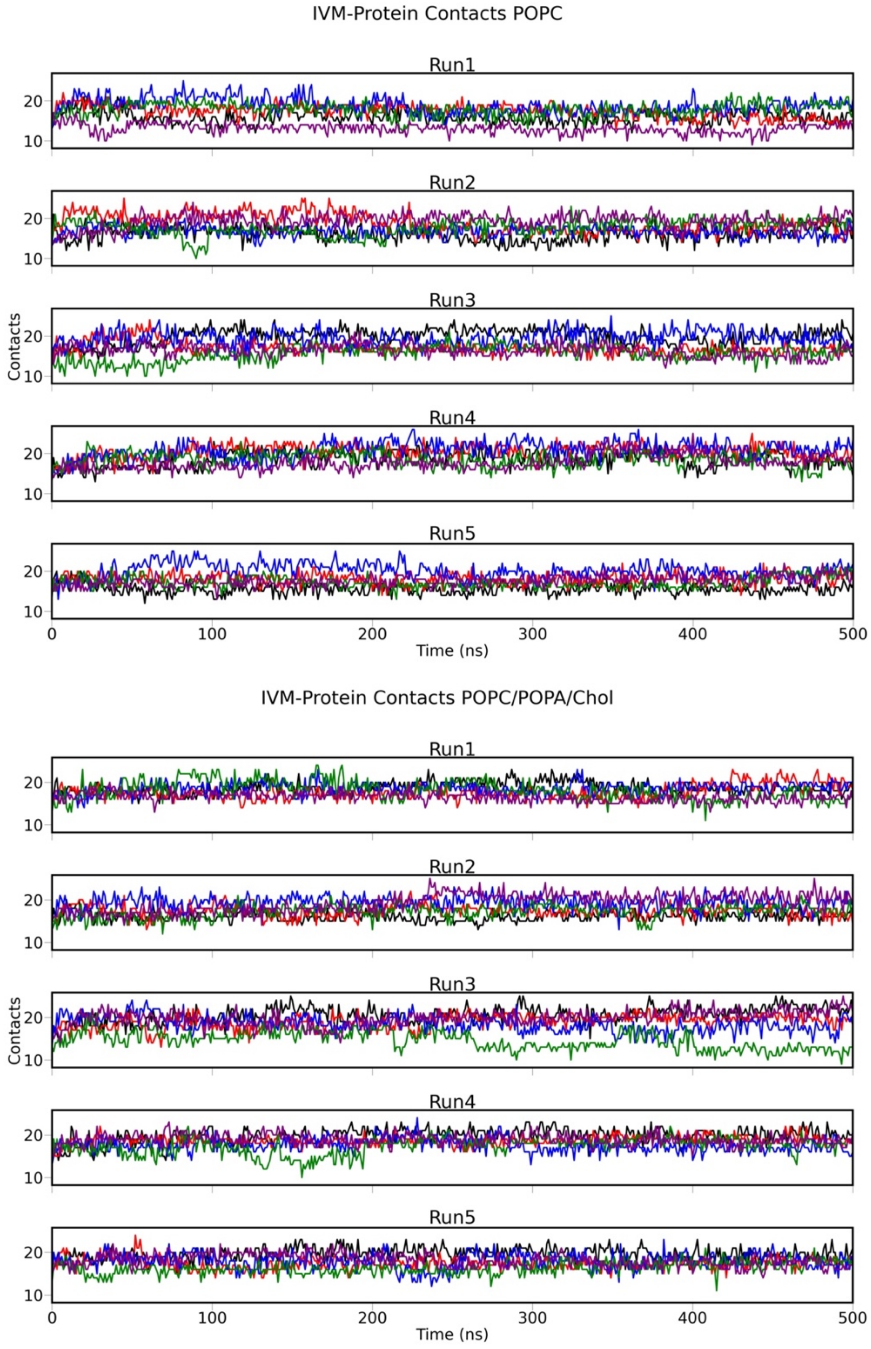

Fig. S12. IVM-protein contacts over the course of MD simulations in two membrane systems, POPC (top) and POPC/POPA/Chol (bottom). Five replications were performed for each system. A contact was counted if the distance between two atoms of IVM and α7nAChR was ≤ 3.5 Å. Each color represents one of five individual IVM molecules in the pentameric α7nAChR TMD+ICD.

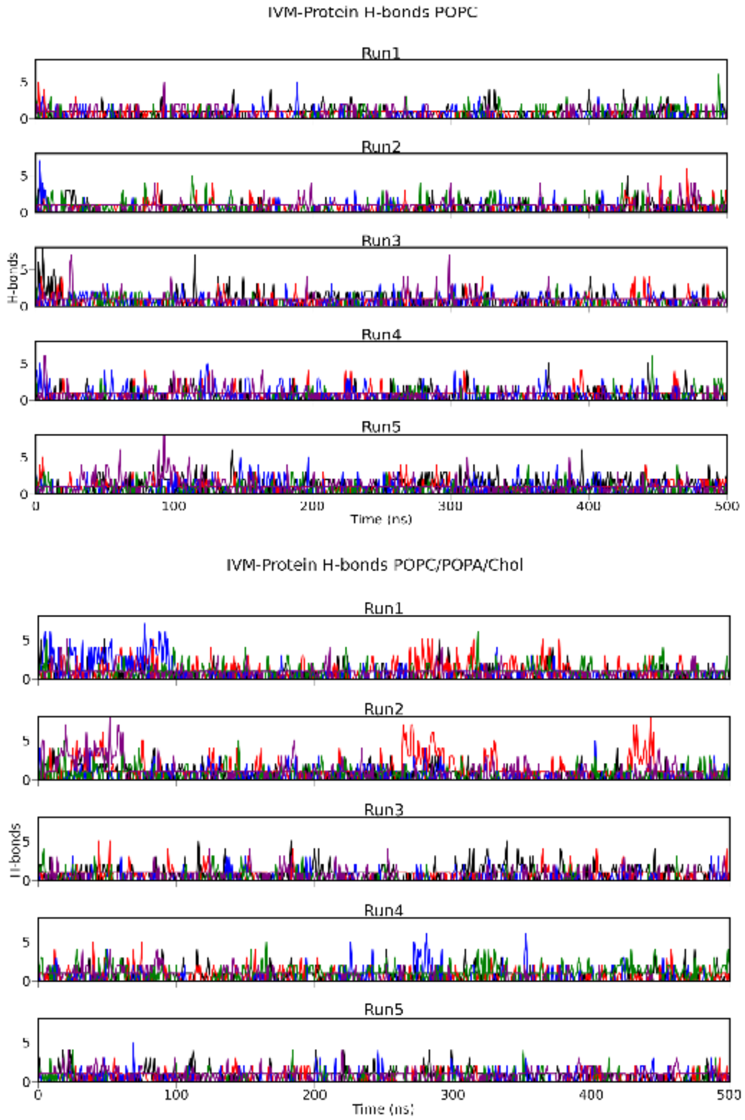

Fig. S13. Hydrogen bonds between IVM and α7nAChR over the course of MD simulations in two membrane systems, POPC (top) and POPC/POPA/Chol (bottom). A hydrogen bond was counted if the distance between the donor (D) and acceptor (A) atoms was ≤ 3.0 Å and the angle of D–H–A was ≥ 120°. At least one hydrogen bond was formed in each of replicating simulations. Each color represents one of five individual IVM molecules in the pentameric α7nAChR TMD+ICD.

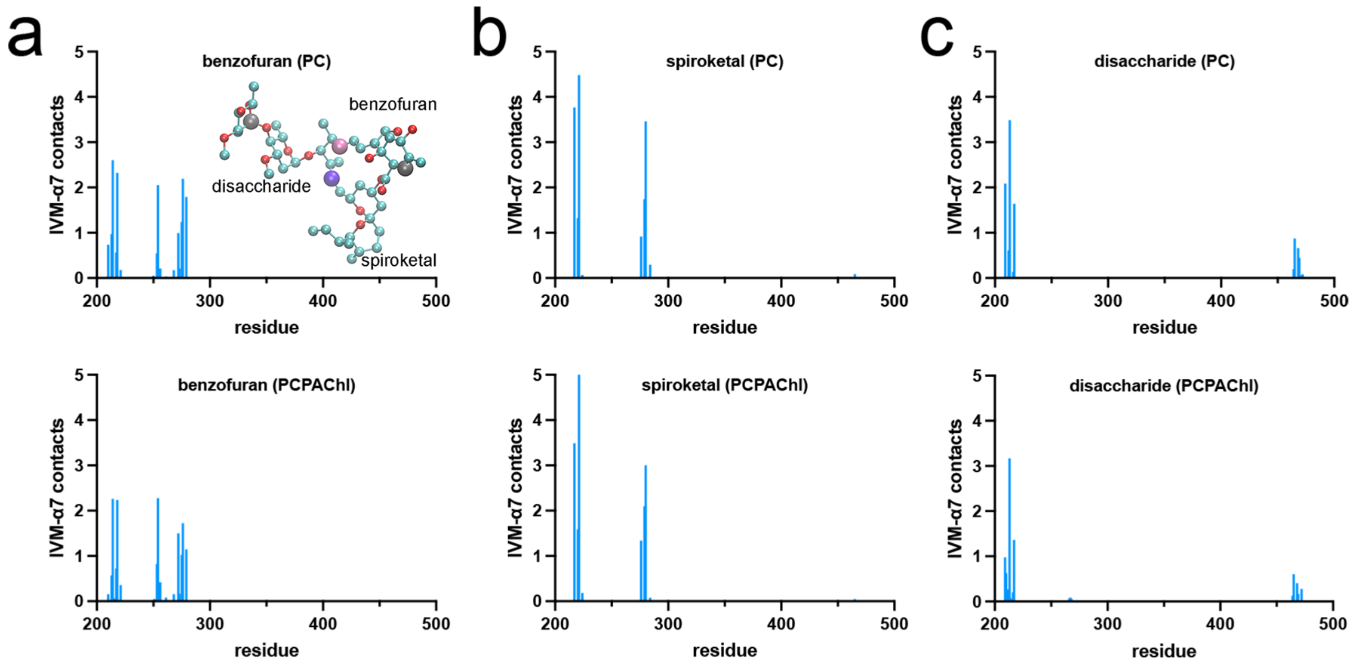

Fig. S14. Average contacts of different IVM moieties to individual residues of α7nAChR in each frame of MD simulations. Contacts were counted separately for (**a**) benzofuran, (**b**) spiroketal, and (**c**) disaccharide moieties of IVM, whose chemical structure is inserted in (a). Contacts in both membrane systems were calculated per frame and monomer and averaged across all the replicates. A contact was counted if two atoms from IVM and α7nAChR are within 3.5 Å. Note that a summation of contacts in the PCPAChl membrane system in (a-c) is shown in Fig. 6b.

**Table S1. Sequence Alignment of IVM-binding Sites**				
**Receptor**	**PDBID**	**TM1**	**TM2**	**TM3**
α7nAChR	8F4V	213**L**NLL**IP**CVL	251TV**FM**LLVA	272A**Q**Y**FA**ST**M**I
α3GlyR	5VDH	225**IQ**MY**IP**SLL	264TTQ**SS**GSR	284**D**IWM**A**VC**LL**
α1GlyR	6VM0 3JAF	249**I**QMY**IP**SL**L**	288**T**TQ**SS**GS**R**	308**D**IWM**A**VC**LL**
GluClR	3RHW	218**L**QLY**IP**SCM	257TAQ**S**AGIN	277**D**VW**IG**AC**MT**
Residues in bold are within 3.0 Å from IVM.				

Table S2. NMR and ESR experimental restraints for α7nAChR structure calculations in a desensitized state (number of restraints per subunit).

NMR NOE distance restraints	820
Intra-residue	472
Inter-residue	348
Sequential ($\|i-j\| = 1$)	254
Medium-range ($2 \leq \|i-j\| \leq 4$)	72
Long-range ($\|i-j\| \geq 5$)	22
Inter-subunit	0
Hydrogen bonds [a]	266
NMR PRE distance restraints [b]	615
Class 1	42
Class 2	297
Class 3	266
Additional inter-subunit [c]	13
DEER ESR distance restraints	18

[a] Hydrogen bond (helical) distance restraints were used for residues 209-231, 238-260, 270-296, 307-321, 361-369, and 409-469.

[b] Class 1, 2, and 3 of PRE distance restraints are defined as follows:
Class 1: $r \leq 16$ Å ($I/I_0 \leq 0.15$)
Class 2: $16 < r < 26$ Å ($0.15 < I/I_0 < 0.85$)
Class 3: $r \geq 26$ Å ($I/I_0 \geq 0.85$)
I and I_0 are NMR peak intensities in paramagnetic and diamagnetic states, respectively.

[c] Including two ^{19}F NMR distance restraints from our study published previously[10].

[d] 3- and 9-residues structural fragments and 14 DEER restraints obtained previously for the α7nAChR TMD+ICD structure calculations[4] were also included in the current calculations.

Table S3. Statistics for the 15 refined α7nAChR structures in a desensitized state.

**Structure statistics** [a]	
Q-factors	
Q^{DEER}	0.12 ± 0.03
Q^{NOE}	0.15 ± 0.16
Q^{PRE}	0.18 ± 0.08
Q^{PRE}_{free} [b]	0.13 ± 0.04
Violations	
NMR NOE distance restraints (Å)	0.20 ± 0.21
NMR PRE distance restraints (Å)	1.15 ± 0.51
DEER ESR distance restraints (Å)	2.12 ± 0.45
Deviations from idealized geometry	
Bond lengths (Å)	0.0036 ± 0.0001
Bond angles (°)	0.92 ± 0.01
Rotamer Outliers (%)	0.22 ± 0.18
Clash score	1.1 ± 0.4
MolProbity score	0.80 ± 0.08
Ramachandran plot	
favored (%)	98.9 ± 0.5
outliers (%)	0.00 ± 0.00
Average pairwise r.m.s. deviation (Å)	
Heavy:	
(Residues 209-472; all residues)	2.65 ± 0.91
(Residues 209-231, 238-260, 270-296, 308-321, 409-469; helical regions)	1.77 ± 0.52
Backbone	
(Residues 209-472; all residues)	2.05 ± 0.67
(Residues 209-231, 238-260, 270-296, 308-321, 409-469; helical regions)	1.26 ± 0.39

[a] The reported are Mean ± SD. Structure statistics were from 15 structures deposited in PDB. Phenix[11] and MolProbity[12] were used for structure statistics.

[b] 56 PRE restraints (~10% of total PRE restraints) that were excluded in structure calculations were used for the Q^{PRE}_{free} calculation.

Table S4. NMR samples, experiments, and most relevant acquisition parameters

Titration NMR experiments to determine ivermectin binding site (determination of changes in α7 TMD-ICD backbone ^1H_N and ^{15}N chemical shifts and intensity of ^1H-^{15}N cross-peaks induced by ivermectin)					
**NMR Experiment**	**Spectral windows**	**Data points in time domain**	**Number of scans**	**Data collection time**	**Other notes**
**Sample 1**: 0.25 mM ^{15}N-labeled α7nAChR C317 TMD-ICD, 5 mM sodium acetate pH4.7, 35 mM (0.8%) LDAO, 25 mM NaCl; magnetic field: 18.8 T; temperature: 318 K					
2D ^1H-^{15}N TROSY-HSQC	13×23 ppm (^1H_N×^{15}N)	2048×160 (^1H_N×^{15}N)	32	2 h	0 mM ivermectin
2D ^1H-^{15}N TROSY-HSQC	13×23 ppm (^1H_N×^{15}N)	2048×160 (^1H_N×^{15}N)	32	2 h	0.03 mM ivermectin; 0.1% DMSO
2D ^1H-^{15}N TROSY-HSQC	13×23 ppm (^1H_N×^{15}N)	2048×160 (^1H_N×^{15}N)	32	2 h	0.1 mM ivermectin; 0.3% DMSO
2D ^1H-^{15}N TROSY-HSQC	13×23 ppm (^1H_N×^{15}N)	2048×160 (^1H_N×^{15}N)	32	2 h	0.25 mM ivermectin; 0.55% DMSO
2D ^1H-^{15}N TROSY-HSQC	13×23 ppm (^1H_N×^{15}N)	2048×160 (^1H_N×^{15}N)	32	2 h	0.5 mM ivermectin; 0.95% DMSO
1D and 2D Saturation Transfer Difference (STD) NMR experiments to determine ivermectin binding site					
**Sample 2-4**: 0.25 mM ^{15}N-labeled α7nAChR C317 TMD-ICD, 5 mM sodium acetate pH4.7, 35 mM (0.8%) LDAO, 25 mM NaCl, 0.8% DMSO, 0.5 mM ivermectin; magnetic field: 18.8 T; temperature: 318 K (saturation of ivermectin peak C1''H/C3H, C11H, or C15H for 2 s; relaxation delay: 3 s)					
1D ^1H STD	16 ppm	16384	8	1 min	Saturation on/off; C1''H/C3H
1D ^1H STD	16 ppm	16384	8	1 min	Saturation on/off; C11H

1D ^{1}H STD	16 ppm	16384	8	1 min	Saturation on/off; C15H
2D ^{1}H-^{15}N STD TROSY-HSQC	13×23 ppm (^{1}H$_{N}$×^{15}N)	2048×160 (^{1}H$_{N}$×^{15}N)	32	11 h	Saturation on/off; C1''H/C3H
2D ^{1}H-^{15}N STD TROSY-HSQC	13×23 ppm (^{1}H$_{N}$×^{15}N)	2048×160 (^{1}H$_{N}$×^{15}N)	32	11 h	Saturation on/off; C11H
2D ^{1}H-^{15}N STD TROSY-HSQC	13×23 ppm (^{1}H$_{N}$×^{15}N)	2048×160 (^{1}H$_{N}$×^{15}N)	32	11 h	Saturation on/off; C15H
2D ^{1}H homonuclear NOESY to determine ivermectin binding site					
**Sample 5**: 0.2 mM ^{15}N-labeled α7nAChR C317 TMD-ICD, 5 mM sodium acetate pH4.7, 35 mM (0.8%) LDAO, 25 mM NaCl, 0.8% DMSO, 0.5 mM ivermectin; magnetic field: 18.8 T; temperature: 318 K					
2D ^{1}H NOESY	14×14 ppm (^{1}H×^{1}H)	2048×400 (^{1}H×^{1}H)	48	7 h	mixing time: 120 ms
2D ^{1}H NOESY	14×14 ppm (^{1}H×^{1}H)	2048×400 (^{1}H×^{1}H)	48	7 h	mixing time: 200 ms
Paramagnetic Relaxation Enhancement (PRE) NMR experiments for determination of α7nAChR TMD-ICD structure in an ivermectin-induced desensitized state by Rosetta					
**Sample 6-7**: 0.22 mM ^{15}N-labeled, V311C-MTSL-labeled α7nAChR TMD-ICD, 5 mM sodium acetate pH4.7, 35 mM (0.8%) LDAO, 25 mM NaCl, 0.4% DMSO (0 and 0.22 mM ivermectin); magnetic field: 18.8 T; temperature: 318 K					
2D ^{1}H-^{15}N TROSY-HSQC	13×23 ppm (^{1}H$_{N}$×^{15}N)	2048×176 (^{1}H$_{N}$×^{15}N)	64	4 h	0 mM ivermectin 0 and 2.2 mM ascorbic acid
2D ^{1}H-^{15}N TROSY-HSQC	13×23 ppm (^{1}H$_{N}$×^{15}N)	2048×176 (^{1}H$_{N}$×^{15}N)	64	4 h	0.22 mM ivermectin 0 and 2.2 mM ascorbic acid
**Sample 8-9**: 0.22 mM ^{15}N-labeled, C335-MTSL-labeled α7nAChR TMD-ICD, 5 mM sodium acetate pH4.7, 26 mM (0.6%) LDAO, 25 mM NaCl, 0.4% DMSO (0 and 0.22 mM ivermectin); magnetic field: 18.8 T; temperature: 318 K					

2D ^1H-^{15}N TROSY-HSQC	13×23 ppm (^1H_N×^{15}N)	2048×176 (^1H_N×^{15}N)	40	2.5 h	0 mM ivermectin 0 and 2.2 mM ascorbic acid
2D ^1H-^{15}N TROSY-HSQC	13×23 ppm (^1H_N×^{15}N)	2048×176 (^1H_N×^{15}N)	40	2.5 h	0.22 mM ivermectin 0 and 2.2 mM ascorbic acid
**Sample 10-11**: 0.2 mM ^{15}N-labeled, C342-MTSL-labeled α7nAChR TMD-ICD, 5 mM sodium acetate pH4.7, 26 mM (0.6%) LDAO, 25 mM NaCl, 0.4% DMSO (0 and 0.2 mM ivermectin); magnetic field: 18.8 T; temperature: 318 K					
2D ^1H-^{15}N TROSY-HSQC	13×23 ppm (^1H_N×^{15}N)	2048×176 (^1H_N×^{15}N)	64	4 h	0 mM ivermectin 0 and 2.0 mM ascorbic acid
2D ^1H-^{15}N TROSY-HSQC	13×23 ppm (^1H_N×^{15}N)	2048×176 (^1H_N×^{15}N)	64	4 h	0.2 mM ivermectin 0 and 2.0 mM ascorbic acid
**Sample 12-13**: 0.2 mM ^{15}N-labeled, S350C-MTSL-labeled α7nAChR TMD-ICD, 5 mM sodium acetate pH4.7, 44 mM (1.0%) LDAO, 25 mM NaCl, 0.4% DMSO (0 and 0.2 mM ivermectin); magnetic field: 18.8 T; temperature: 318 K					
2D ^1H-^{15}N TROSY-HSQC	13×23 ppm (^1H_N×^{15}N)	2048×176 (^1H_N×^{15}N)	96	7 h	0 mM ivermectin 0 and 2.0 mM ascorbic acid
2D ^1H-^{15}N TROSY-HSQC	13×23 ppm (^1H_N×^{15}N)	2048×176 (^1H_N×^{15}N)	96	7 h	0.2 mM ivermectin 0 and 2.0 mM ascorbic acid
**Sample 14-15**: 0.20 mM ^{15}N-labeled, C375-MTSL-labeled α7nAChR TMD-ICD, 5 mM sodium acetate pH4.7, 35 mM (0.8%) LDAO, 20 mM NaCl, 0.4% DMSO (0 and 0.2 mM ivermectin); magnetic field: 18.8 T; temperature: 318 K					
2D ^1H-^{15}N TROSY-HSQC	13×23 ppm (^1H_N×^{15}N)	2048×176 (^1H_N×^{15}N)	64	4 h	0 mM ivermectin

					0 and 2.0 mM ascorbic acid
2D ^{1}H-^{15}N TROSY-HSQC	13×23 ppm ($^{1}H_N$×^{15}N)	2048×176 ($^{1}H_N$×^{15}N)	64	4 h	0.2 mM ivermectin 0 and 2.0 mM ascorbic acid
**Sample 16-17**: 0.25 mM ^{15}N-labeled, C390-MTSL-labeled α7nAChR TMD-ICD, 5 mM sodium acetate pH4.7, 44 mM (1.0%) LDAO, 25 mM NaCl, 0.4% DMSO (0 and 0.25 mM ivermectin); magnetic field: 18.8 T; temperature: 318 K					
2D ^{1}H-^{15}N TROSY-HSQC	13×23 ppm ($^{1}H_N$×^{15}N)	2048×176 ($^{1}H_N$×^{15}N)	56	3.5 h	0 mM ivermectin 0 and 2.5 mM ascorbic acid
2D ^{1}H-^{15}N TROSY-HSQC	13×23 ppm ($^{1}H_N$×^{15}N)	2048×176 ($^{1}H_N$×^{15}N)	56	3.5 h	0.25 mM ivermectin 0 and 2.5 mM ascorbic acid
**Sample 18-19**: 0.24 mM ^{15}N-labeled, L415C-MTSL-labeled α7nAChR TMD-ICD, 5 mM sodium acetate pH4.7, 35 mM (0.8%) LDAO, 25 mM NaCl, 0.4% DMSO (0 and 0.24 mM ivermectin); magnetic field: 18.8 T; temperature: 318 K					
2D ^{1}H-^{15}N TROSY-HSQC	13×23 ppm ($^{1}H_N$×^{15}N)	2048×176 ($^{1}H_N$×^{15}N)	64	4 h	0 mM ivermectin 0 and 2.4 mM ascorbic acid
2D ^{1}H-^{15}N TROSY-HSQC	13×23 ppm ($^{1}H_N$×^{15}N)	2048×176 ($^{1}H_N$×^{15}N)	64	4 h	0.24 mM ivermectin 0 and 2.4 mM ascorbic acid
**Sample 20-21**: 0.20 mM ^{15}N-labeled, C435-MTSL-labeled α7nAChR TMD-ICD, 5 mM sodium acetate pH4.7, 22 mM (0.5%) LDAO, 25 mM NaCl, 0.4% DMSO (0 and 0.2 mM ivermectin); magnetic field: 18.8 T; temperature: 318 K					
2D ^{1}H-^{15}N TROSY-HSQC	13×23 ppm ($^{1}H_N$×^{15}N)	2048×176 ($^{1}H_N$×^{15}N)	56	3.5 h	0 mM ivermectin 0 and 2.0 mM ascorbic acid

2D ^{1}H-^{15}N TROSY-HSQC	13×23 ppm (^1H_N×^{15}N)	2048×176 (^1H_N×^{15}N)	56	3.5 h	0.2 mM ivermectin 0 and 2.0 mM ascorbic acid
3D HNCO to determine ligand binding site					
**Sample 22-23**: 0.20 mM ^{15}N-labeled α7nAChR C317 TMD-ICD, 5 mM sodium acetate pH4.7, 44 mM (1.0%) LDAO, 25 mM NaCl; magnetic field: 18.8 T; temperature: 318 K					
3D TROSY-HNCO	12×23×12 ppm (^1H_N×^{15}N×^{13}C)	1024×48×56 (^1H_N×^{15}N×^{13}C)	16	14 h	0 mM ivermectin and PNU
3D TROSY-HNCO	12×23×12 ppm (^1H_N×^{15}N×^{13}C)	1024×48×56 (^1H_N×^{15}N×^{13}C)	16	14 h	0.03 mM ivermectin and PNU 0.2% DMSO
Assignment of ^{1}H chemical shifts of ivermectin					
**Sample 24**: 1 mM ivermectin, 5 mM sodium acetate pH4.7, 35 mM (0.8%) LDAO, 25 mM NaCl, 1.6 % DMSO; magnetic field: 14.1 T; temperature: 318 K					
2D ^{1}H NOESY	10×10 ppm (^{1}H×^{1}H)	1024×256 (^{1}H×^{1}H)	16	2 h	NOESY mixing time: 250 ms
2D ^{1}H TOCSY	10×10 ppm (^{1}H×^{1}H)	1024×256 (^{1}H×^{1}H)	16	2 h	TOCSY mixing time: 60 ms

*A recycle delay (D1) of 1 s was used in all NMR experiments, except 1D and 2D STD NMR experiments (3 s).
All samples contained 20 μM DSS for the reference of the 0 ppm ^{1}H chemical shift.

References

1. Huang, X.; Chen, H.; Shaffer, P. L., Crystal Structures of Human GlyRalpha3 Bound to Ivermectin. *Structure* **2017,** *25*, 945-950 e2.
2. Clore, G. M.; Tang, C.; Iwahara, J., Elucidating transient macromolecular interactions using paramagnetic relaxation enhancement. *Curr Opin Struct Biol* **2007,** *17*, 603-16.
3. Stein, R. A.; Beth, A. H.; Hustedt, E. J., A Straightforward Approach to the Analysis of Double Electron-Electron Resonance Data. *Methods Enzymol* **2015,** *563*, 531-67.
4. Bondarenko, V.; Wells, M. M.; Chen, Q.; Tillman, T. S.; Singewald, K.; Lawless, M. J.; Caporoso, J.; Brandon, N.; Coleman, J. A.; Saxena, S.; Lindahl, E.; Xu, Y.; Tang, P., Structures of highly flexible intracellular domain of human alpha7 nicotinic acetylcholine receptor. *Nat Commun* **2022,** *13*, 793.
5. Noviello, C. M.; Gharpure, A.; Mukhtasimova, N.; Cabuco, R.; Baxter, L.; Borek, D.; Sine, S. M.; Hibbs, R. E., Structure and gating mechanism of the alpha7 nicotinic acetylcholine receptor. *Cell* **2021**.
6. Zhao, Y.; Liu, S.; Zhou, Y.; Zhang, M.; Chen, H.; Eric Xu, H.; Sun, D.; Liu, L.; Tian, C., Structural basis of human alpha7 nicotinic acetylcholine receptor activation. *Cell Res* **2021,** *31*, 713-716.
7. Gharpure, A.; Teng, J.; Zhuang, Y.; Noviello, C. M.; Walsh, R. M., Jr.; Cabuco, R.; Howard, R. J.; Zaveri, N. T.; Lindahl, E.; Hibbs, R. E., Agonist Selectivity and Ion Permeation in the alpha3beta4 Ganglionic Nicotinic Receptor. *Neuron* **2019,** *104*, 501-511 e6.
8. Basak, S.; Gicheru, Y.; Rao, S.; Sansom, M. S. P.; Chakrapani, S., Cryo-EM reveals two distinct serotonin-bound conformations of full-length 5-HT3A receptor. *Nature* **2018,** *563*, 270-274.
9. Smart, O. S.; Neduvelil, J. G.; Wang, X.; Wallace, B. A.; Sansom, M. S., HOLE: a program for the analysis of the pore dimensions of ion channel structural models. *J Mol Graph* **1996,** *14*, 354-60, 376.
10. Bondarenko, V.; Wells, M. M.; Chen, Q.; Singewald, K. C.; Saxena, S.; Xu, Y.; Tang, P., (19)F Paramagnetic Relaxation-Based NMR for Quaternary Structural Restraints of Ion Channels. *ACS Chem Biol* **2019,** *14*, 2160-2165.
11. Adams, P. D.; Afonine, P. V.; Bunkoczi, G.; Chen, V. B.; Davis, I. W.; Echols, N.; Headd, J. J.; Hung, L. W.; Kapral, G. J.; Grosse-Kunstleve, R. W.; McCoy, A. J.; Moriarty, N. W.; Oeffner, R.; Read, R. J.; Richardson, D. C.; Richardson, J. S.; Terwilliger, T. C.; Zwart, P. H., PHENIX: a comprehensive Python-based system for macromolecular structure solution. *Acta Crystallogr D Biol Crystallogr* **2010,** *66*, 213-21.
12. Chen, V. B.; Arendall, W. B., 3rd; Headd, J. J.; Keedy, D. A.; Immormino, R. M.; Kapral, G. J.; Murray, L. W.; Richardson, J. S.; Richardson, D. C., MolProbity: all-atom structure validation for macromolecular crystallography. *Acta Crystallogr D Biol Crystallogr* **2010,** *66*, 12-21.
